# Brain architecture-based vulnerability to traumatic injury

**DOI:** 10.3389/fbioe.2022.936082

**Published:** 2022-08-24

**Authors:** Jared A. Rifkin, Taotao Wu, Adam C. Rayfield, Erin D. Anderson, Matthew B. Panzer, David F. Meaney

**Affiliations:** ^1^ Department of Bioengineering, University of Pennsylvania, Philadelphia, PA, United States; ^2^ Department of Mechanical and Aerospace Engineering, University of Virginia, Charlottesville, VA, United States; ^3^ Department of Biomedical Engineering, University of Virginia, Charlottesville, VA, United States; ^4^ Department of Neurosurgery, University of Pennsylvania, Philadelphia, PA, United States

**Keywords:** Kuramoto model, structural connectivity, brain networks, traumatic brain injury, lesions

## Abstract

The white matter tracts forming the intricate wiring of the brain are subject-specific; this heterogeneity can complicate studies of brain function and disease. Here we collapse tractography data from the Human Connectome Project (HCP) into structural connectivity (SC) matrices and identify groups of similarly wired brains from both sexes. To characterize the significance of these architectural groupings, we examined how similarly wired brains led to distinct groupings of neural activity dynamics estimated with Kuramoto oscillator models (KMs). We then lesioned our networks to simulate traumatic brain injury (TBI) and finally we tested whether these distinct architecture groups’ dynamics exhibited differing responses to simulated TBI. At each of these levels we found that brain structure, simulated dynamics, and injury susceptibility were all related to brain grouping. We found four primary brain architecture groupings (two male and two female), with similar architectures appearing across both sexes. Among these groupings of brain structure, two architecture types were significantly more vulnerable than the remaining two architecture types to lesions. These groups suggest that mesoscale brain architecture types exist, and these architectural differences may contribute to differential risks to TBI and clinical outcomes across the population.

## Introduction

Despite growing public awareness, traumatic brain injury (TBI) continues to be a significant health issue responsible for nearly three million emergency room visits annually in the United States (Taylor et al., 2017). The cost of TBI in the United States is $40.6 billion per year and TBI is on pace to become the third leading cause of death worldwide by 2025 (Miller et al., 2021). TBI can occur from a wide variety of everyday activities, such as contact sports, involvement in a vehicular collision, or even just falls from a standing height, demonstrating that an otherwise typically healthy population is at risk for TBI (Asemota et al., 2013). Therefore, understanding the mechanisms of TBI is key to developing protective headgear and mitigating its great socioeconomic burdens across a large population.

Computational biomechanics is a key tool used by researchers to mitigate the consequences of TBI, and its role in developing protective headgear and passive safety equipment in motor vehicles is well-documented (Fredriksson et al., 2011; Dymek et al., 2021). Finite element (FE) analysis is frequently leveraged (Kleiven and von Holst, 2002; Mao et al., 2013; Gabler et al., 2016) to relate impact conditions to the movement and deformation of the brain within the skull, with recent models developed specifically for improving helmet design (Kurt et al., 2017; Giudice et al., 2020b). In recent years, there has been a growth in interdisciplinary models that integrate both the mechanics of brain movement and the corresponding changes to the brain’s axonal network structure (Kraft et al., 2012; Sullivan et al., 2015; Giordano et al., 2017; Wu et al., 2019; Giudice et al., 2020a; Hajiaghamemar et al., 2020). A common approach in these studies is to embed the structural connectivity (SC) network of a brain, representing the connections among grey matter regions and white matter tracts (Bassett and Sporns, 2017), into the FE brain models and predict how information transfer paths in the brain would change from the impact. As the FE models improve—e.g., by incorporating the anisotropic material properties of the model—these interdisciplinary methods will provide a more direct comparison between white matter concentrations and the strain time-history of a corresponding region in the FE model. Brain deformation (strain) can be outputted from these FE models and strain injury criteria can then be implemented to predict injury severity (Bain and Meaney, 2000; Wright and Ramesh, 2012; Wu et al., 2021, 2022b). Together, it is now possible for helmet designers to identify regions of the brain that experience the greatest magnitude of deformation, estimate how this can affect or injure cellular and subcellular structure, and estimate an overall injury risk (Fanton et al., 2019; Anderson et al., 2020).

Until now, many of these computational models examining TBI risk rarely examined a range of sizes that could include both male and female subjects or considered a wide range of brain shapes (Reynier et al., 2021). Despite females exhibiting a greater risk of experiencing TBI (van Pelt et al., 2019; Bretzin et al., 2021), biomechanical computational models of TBI are historically derived from male anatomies and kinematics. The exact mechanism for a sex-specific risk factor could emerge at the ultrastructural levels, where there are key differences in the microtubule network (Benice et al., 2006; Dollé et al., 2018). Alternatively, the differences could appear at the brain architecture level, where sex-dependent changes in the SC network could lead to a differential risk of injury from impact between males and females, or within subgroups (Xin et al., 2019).

Collectively, these factors point to an opportunity for examining whether differential risks exist among subgroups within the population. Two potential sources of risk difference associated with the biomechanics of TBI can occur from variations in the physical characteristics of the brain (e.g., size, shape, material properties) and variations of the brain architecture. In the most detailed form, this would lead to asking whether TBI risk is subject-specific (Anderson et al., 2020), which could have important implications for individual risk to an impact and point towards personalized head protection strategies. Some current methods already approach the possible differential risks for injury associated with a range of physical properties that include brain size and shape. For instance, image registration can be used to rapidly generate individual-specific FE brain models by morphing a template brain model into one that matches the anatomy of a subject (Giudice et al., 2020a). In this case, the mechanics of TBI are simulated with a model that is individual-specific in anatomy, but this process currently does not consider the aspects of the individual’s brain neuronal networks, a feature that may provide more direct insight into the neurological impairment that could occur from an impact (Sharp et al., 2014; Hellyer et al., 2015; Váša et al., 2015; Gilbert et al., 2018). The possibility that impacts may differentially map onto different brain architectures is supported by clinical imaging data that show even simple features of network architecture and information flow will change because of TBI, and that the extent of these changes are correlated to patient outcome (Caeyenberghs et al., 2014; Yuan et al., 2015; Dall’Acqua et al., 2017; van der Horn et al., 2017). However, these clinical studies do not provide any information regarding the biomechanics of a specific impact, thereby limiting the ability to draw a more direct connection between impact and network dynamics.

Compared to other approaches used to model brain and neuronal dynamics, Kuramoto oscillator models (KMs) are well suited to leverage the information from a network architecture in their simulations. Many models of neural activity such as Hodgkin-Huxley or the Izihkevich integrate-and-fire model, represent the activities and membrane potentials of individual neurons (Hodgkin and Huxley, 1952; Izhikevich, 2003). KMs instead model the collective activity of neuronal masses and can be used to study global dynamics. Other comparable models of global dynamics include Fitzhugh-Nagumo, Wilson-Cowan, neural-mass, and spiking neurons; however, the KM offers a simple model with relatively few parameters without sacrificing predictive ability (Messé et al., 2015). KMs are a class of coupled oscillator models that use the edge strength in the brain SC network to predict how the oscillatory behavior of neuronal activity in different brain regions affect each other (Cumin and Unsworth, 2007; Cabral et al., 2011; Schmidt et al., 2015; Siettos and Starke, 2016; Lee et al., 2017; Fukushima and Sporns, 2018). In combination with FE models of the brain architecture, KMs provide a path to directly examine individual-specific responses to impact and build upon previous models that imply specific lesions in the brain can have a subject-specific difference in their severity (Honey and Sporns, 2008; Váša et al., 2015). By analyzing how metrics of oscillator coalescence (synchrony) and state switching (metastability) change as a function of network lesion, KMs offer analogs to how cognition can be altered (Córdova-Palomera et al., 2017).

Our broad objective in this study was twofold: 1) to determine if there was sufficient similarity among a population of brain architectures to define distinct architecture subgroups among male and female brains, and 2) to study if these distinct architectures would show a differential vulnerability to simulated TBI. Using KMs, we evaluated how structural groupings translate to unique patterns in simulated neural dynamics, asking whether these dynamical groupings would show consistency with the differences observed in the structural network. Our study demonstrates that the neural architecture is not homogenous in the population but can be grouped into distinct subpopulations among male and female subjects. In turn, these structural architectures display a differential risk to lesions that target hub nodes in each architectural group. Together, these results demonstrate the potential role that architecture could play in outcome and lead the way towards future studies which can test if these architecture-based differences play a role in concussion risk after head impact.

## Materials and methods

### Computing structural connectivity from human connectome project Data

Our dataset of structural connectivity (SC) matrices came from the human connectome project (HCP) young adult data (van Essen et al., 2013). As described in Wu et al., 2022b, we computed SC for a subject by examining tractography in the Schaefer 100 parcellation (a resolution of 100 nodes) (Schaefer et al., 2018). We used DSI Studio (http://dsi-studio.labsolver.org) with the following settings for deterministic whole-brain fiber tracking: q-space diffeomophic reconstruction (Yeh and Tseng, 2011), a mean diffusion distance ratio of 1.25, an angular cutoff of 55, a step size of 1.0 mm, a minimum length of 10 mm, a spin density function smoothing of 0.0, a maximum length of 400 mm and a cutoff of 1,000,000 streamlines. This yielded 1,065 networks, of which 490 were male and 575 were female. We scaled the edge weights in these networks by the number of edges such that the mean weight was $\frac{1}{10,000}$ .

### Reparcellation and similarity scores

**FIGURE 1:**
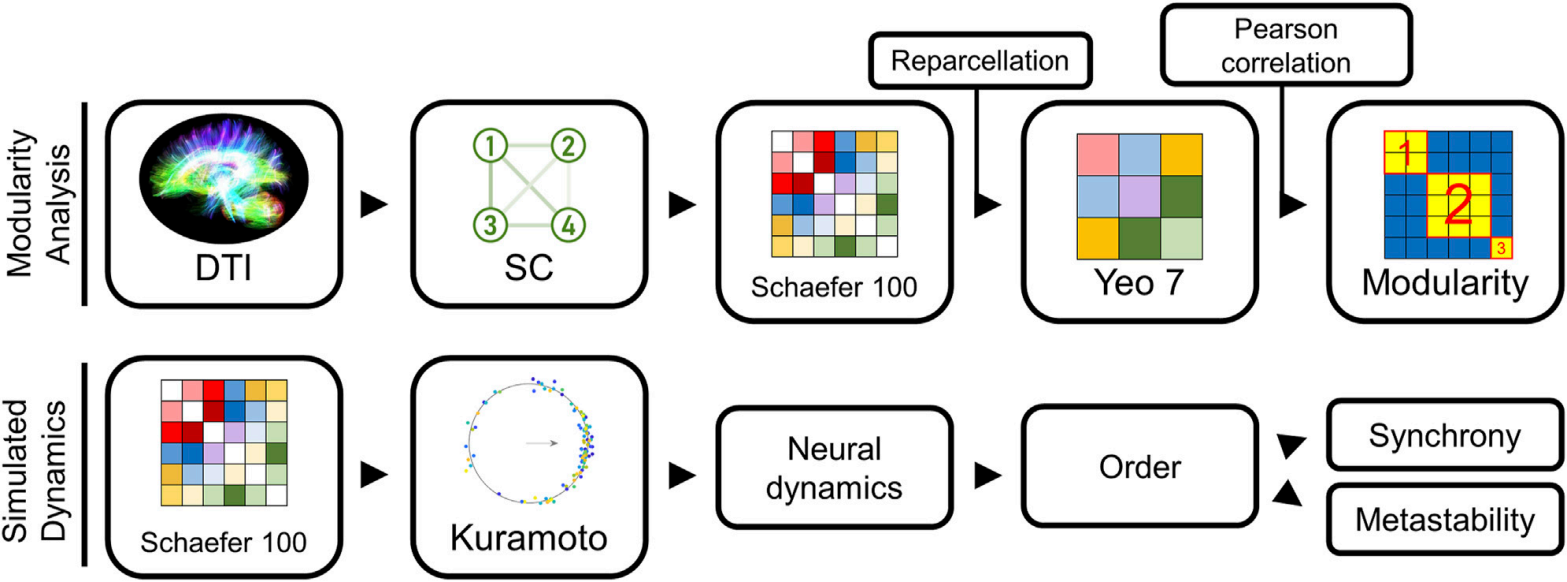
Modeling pipeline overview. Our methodology used two modeling approaches: (1) an approach to group similar structural connectivity (SC) matrices into distinct types, and (2) a method to convert any SC into an estimate of the neural dynamics using a Kuramoto oscillator model (KM). Our first approach examined the different architectural groupings among the SC matrices extracted from the Human Connectome Project dataset (top). To reduce the bias from sparse connectivity matrices on the subsequent groupings, we reparcellated Schaefer 100 into Yeo 7 representations, and then computed Pearson correlation for every pair of Yeo 7 networks in our sample. These pairwise similarity scores were collated in a similarity matrix. Modularity calculations on this similarity matrix identified which networks exhibited the greatest similarity. Our second modeling approach started with the Schaefer 100 SC network for a subject (bottom), used a KM to predict brain activity.

We sought to sort our subjects by using their structural connectivity matrices (Figure 1). To do so, we reshaped our matrices into 1D arrays; each unique SC edge corresponded to a dimension in this vector. For instance, a network generated using the Schaefer 100 parcellation would be reshaped into a 10,000-dimensional vector (4,950 edges come from its upper triangle, the symmetric 4,950 edges in the lower triangle would be removed as 0’s, and the remaining 100 trivial edges would consist of 1’s along the diagonal). These sparsely distributed data lead to difficulty with Euclidean-distance-based clustering methods (Domingos, 2012). Therefore, we reduced the dimensionality further by recalculating the SCs using the Yeo 7 parcellation, which only has 21 informative dimensions. For our work, we computed the mean weight of every Schaefer 100 edge contained within a single Yeo 7 edge, moving through each of the Yeo 7 edges (Yeo et al., 2011; Schaefer et al., 2018). To circumvent any remaining challenges associated with using Euclidean-distance-based measures, we used Pearson correlation to evaluate similarity. A value of 1 indicated perfect similarity, 0 indicated complete dissimilarity, and -1 indicated anticorrelated vectors. For both sexes, we computed a matrix of similarity between each pair of architectures.

### Modularity

After generating similarity matrices, we identified clusters of similar subjects by employing modularity analysis. Modularity is a graph theory metric that is used to highlight and identify groups of nodes that exhibit higher connectivities to each other than nodes not belonging to the group (Newman, 2006; Reichardt and Bornholdt, 2006). We used the Brain Connectivity Toolbox (BCT) and MATLAB (Mathworks Inc., Natick, MA.) to compute the modularity of our similarity matrices (Rubinov and Sporns, 2010). Their algorithm maximizes modularity according to the following equation:

$$Q\left(\gamma \right)=\sum_{jh=1,1}^{n,n}\left[A_{jh}-\gamma P_{jh}\right]\delta \left(\sigma_{j},\sigma_{h}\right),$$

where Q is modularity, γ is resolution, *n* is the number of nodes, $A_{jh}$ is the observed edge between nodes *j* and *h*, $P_{jh}$ is the expected edge weight given the degree of nodes *j* and *h*, and δ is the Kronecker delta function that is 1 if nodes are in the same group and 0 otherwise. Q will be approximately 0 if a network is randomly connected and cannot be organized. To ensure that our modularity method would be able to identify groups consistently among populations of different scale, we optimized γ . The value for γ we chose was the minimum that produced nontrivial groupings (i.e., the first value for which two modules were produced). Subjects were assigned an identifier according to their modules.

To ensure the robustness of our groupings, we repeated the modularity analysis using 5-fold cross-validation for both sexes. For the one fifth of networks withheld from the initial modularity analysis, we identified their modules according to the corresponding mean scores in the similarity matrix. We computed accuracy as the fraction of withheld networks that were placed in their original module from the whole sample analysis. We also examined the significance and sufficiency of two modules, by repeating some of our analyses on the groupings that are yielded with other values of γ . For each module, we measured the distributions of various graph metrics (described in more detail below) and computed the significance of the differences between each group.

### Subset similarity and modularity

Using the Schaefer 100 to Yeo 7 parcellation mapping, we identified all Schaefer 100 edges that existed between two Yeo 7 systems. With these edges, we recomputed male and female Pearson correlation similarity matrices. If system edges were off the diagonal of the Yeo 7 matrix (connections between two distinct systems) we computed the correlation of all Schaefer 100 edges. If only the edges within a single system were considered, we reduced the information to the upper triangle of this submatrix. We repeated modularity and cross-validation on these new similarity matrices. Subset accuracy, much like cross validation accuracy, was the fraction of networks that were assigned to the same module when all Yeo 7 edges were considered, with 80% accuracy being considered ideal.

### Kuramoto oscillators

We employed the Kuramoto oscillator model (KM) to simulate neural dynamics from SCs. The behavior of our KMs was defined by the following system of differential equations:

$$d\theta_{j}\left(t\right)=\omega_{j}+K\sum_{h=1}^{N}C_{jh}\sin\left(\theta_{h}\left(t\right)-\theta_{j}\left(t\right)\right)dt,$$

where $\theta_{j}\left(t\right)$ is the phase of the oscillator *j* at time *t*, $\omega_{j}$ is the oscillator’s intrinsic frequency, *K* is the global coupling strength parameter, *N* is the number of oscillators, and $C_{jh}$ is the connectivity between oscillators *j* and *h* (range 0–1).

For all KM simulations, we assigned the natural frequency ( $\omega_{j}$ ) from a normal distribution centered at 60 Hz with a standard deviation of 1 Hz as in Cabral et al., 2011. Initial phases were randomly sampled from a uniform distribution between 0 and 2 π . Simulations lasted 100 s to ensure a stable solution was reached. We used the structural connectome to weigh connectivity among oscillators. To ensure consistency across connectomes, we used a subject’s Schaefer 100 network and normalized the edges such that the mean of off-diagonal elements was 1.

We computed several outputs from the KM (Cabral et al., 2011). First, the order parameter representing the magnitude of the mean phase was calculated using the equation:

$$r\left(t\right)e^{i\phi \left(t\right)}=\frac{1}{N}\sum_{j=1}^{N}e^{i\theta_{j}\left(t\right)},$$

where *r(t)* is the instantaneous order, ϕ(t) is the instantaneous mean phase, *N* is the total number of oscillators, and $\theta_{j}$ is the phase of oscillator *j* at time *t*. The order parameter measures the instantaneous organization of the system of Kuramoto oscillators. From r, we then calculated synchrony, a measure of the network’s ability to coalesce its oscillators into unified states, as the mean of the order parameter. Metastability, which represents the network’s ability to shift between synchronous states, was the order parameter’s standard deviation.

We also tested a form of the KM that used delay differential equations to incorporate possible signal latency between oscillators. In this preliminary analysis across a limited range of coupling strengths, we noticed that our group-dependent results remained consistent, so we moved forward with the more computationally efficient form described above.

### Targeted lesioning

Our lesioning method was derived from (Alstott et al., 2009), whose approach removed all connections from the node with the highest degree, computed the resulting graph theory metrics of the remaining network, and repeated the deletion process until no nodes remained in the SC network. We used node degree to rank the relative connectivity of each node to other nodes within the network to create a ranked list for each brain architecture group, listing nodes in descending degree rank. To map this method—which was applied to a single SC network—onto our population of multiple SC networks, we averaged the nodes’ rankings in every subject. This strategy ensured that we would consistently delete the same nodes across all networks. We also limited our lesions to nodes within the top 5th, 10th, 25th, 50th, and 75th percentile rank. After each lesioning step, we repeated our earlier analyses by simulating the dynamics of the injured SC networks with the KMs. We compared the synchrony and metastability from the KMs as well as various graph metrics of the resulting SC matrices after each deletion.

### Graph metrics

We computed several graph metrics to characterize our SC matrices. Unless specified, all MATLAB functions were from the BCT (Rubinov and Sporns, 2010). Global efficiency was computed as: $GE=\frac{1}{n}\sum_{j=1}^{n}\frac{\sum_{h=1}^{n}d_{jh}^{-1}}{n-1},j\neq h$ where *GE* is global efficiency, *n* is the number of nodes, and $d_{jh}$ is the shortest path between nodes *j* and *h*. Mean shortest path length was the characteristic path length computed as: $L=\frac{1}{n}\sum_{j=1}^{N}\frac{\sum_{h=1}^{N}d_{jh}}{n-1},j\neq h$ with the same variables as global efficiency in addition to *L* representing mean shortest path length. Betweenness centrality for a node was: $BC_{j}=\frac{1}{\left(n-1\right)\left(n-2\right)}\sum_{g=1}^{n}\sum_{h=1}^{n}\frac{\rho_{gh}\left(j\right)}{\rho_{gh}},\;g\neq h,g\neq j,\;h\neq j,$ where $BC_{j}$ is the betweenness centrality for node *j*, *n* is the number of nodes, $\frac{\rho_{gh}\left(j\right)}{\rho_{gh}}$ is the fraction of shortest paths between nodes *g* and *h* that include node *j*. We computed the mean betweenness centrality of all nodes in a network. Clustering coefficient for a node was: $CC_{j}=\frac{1}{n}\sum_{j=1}^{n}\frac{2t_{j}}{k_{j}\left(k_{j}-1\right)},$ where $CC_{j}$ is the clustering coefficient of node *j*, *n* is the number of nodes, $t_{j}$ is the number of triangles around node *j*, and $k_{j}$ is the degree of node *j*.

### Statistical analysis

Unless otherwise noted, all statistical analyses were performed at α = 0.05. We verified normality via visual inspection of quantile-quantile plot linearity. We compared multiple distributions using one-way ANOVA and module assignment as the categorical factor, correcting for multiple comparisons using the Tukey-Kramer method. Unless otherwise noted, error bars for sample-based distributions represent standard error.

## Results

### Types of architectures could be identified within populations of brain networks

**FIGURE 2:**
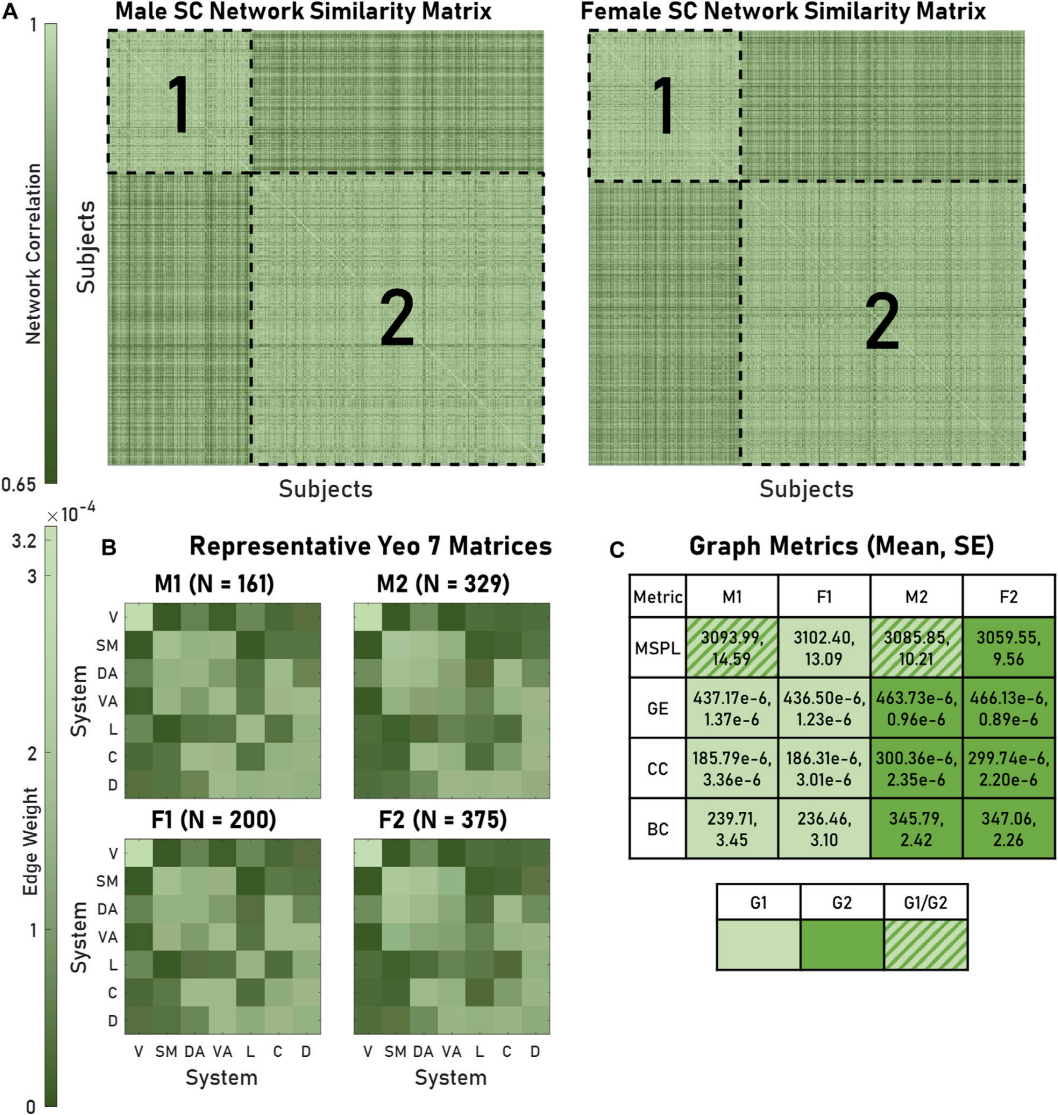
Modules of brain networks with similar architectures exhibited distinct structural graph metrics. **(A)** As visualized in the similarity matrices for both male and female structural connectivity (SC) networks, four modules (outlined in dashed lines) appeared. Within a module similarity was greater than outside (0.942 and 0.906 respective mean correlations). **(B)** The mean Yeo 7 edge weights for all networks within a module produced representative matrices. **(C)** SC metrics (MSPL, GE, CC, BC) produced module-dependent distributions (for a given row, cells containing the same color did not significantly differ). A key is also provided. For GE, CC, and BC, M1-F1 and M2-F2 modules produced significant pairings. Abbreviations used: M1, Male 1; M2, Male 2; F1, Female 1; F2, Female 2; G1, Group 1; G2, Group 2; V, Visual; SM, Somatomotor; DA, Dorsal Attention; VA, Ventral Attention; L, Limbic; C, Control; D, Default; MSPL, Mean shortest path length; GE, global efficiency; CC, Clustering coefficient; BC, Betweenness centrality.

We used modularity to identify groups of brains that exhibited similar architectures, finding two modules for both males and females. For every brain, the average intramodule correlation was significantly greater than the average intermodule correlation: 0.942 and 0.906, respectively (*p* < 0.05) (Figure 2A). Hereafter, we refer to these modules as Male 1 (M1), Male 2 (M2), Female 1 (F1), and Female 2 (F2). We generated representative structural connectivity (SC) matrices by computing each edge’s mean weight among all brains assigned to that particular module (Figure 2B). When comparing the graph metrics of the brains using module assignment as the categorical factor, we saw the appearance of two statistically distinguishable pairs: M1 and F1 networks had significantly different global efficiencies (GEs), clustering coefficients (CCs), and betweenness centralities (BCs), from M2 and F2, but neither M1 compared to F1 nor M2 compared to F2 were significantly different (Figure 2C). When considering mean shortest path length, M1 and F1 had dissimilar distributions.

### Two modules were sufficient for identifying brain architecture types

**FIGURE 3:**
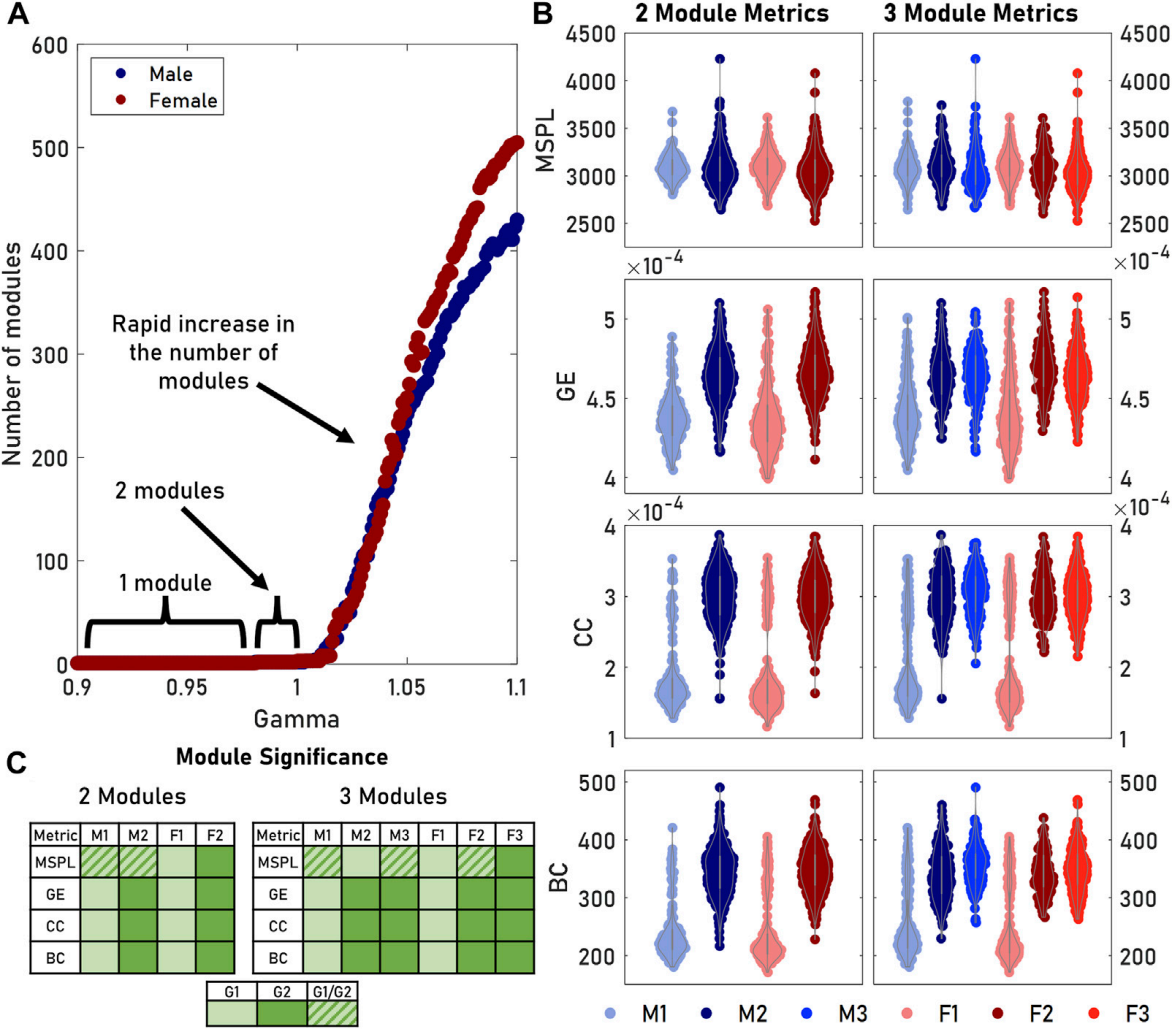
Tuning the gamma parameter in the modularity algorithm produced differing numbers of modules. **(A)** The number of modules remained relatively stable at gamma values below 1. Increasing gamma allowed us to detect more—but smaller—modules. **(B)** We measured the distributions of various graph metrics (MSPL, GE, CC, BC) for both two and three male and female modules. The third module was comparable to the second module for both sexes, while M1 and F1 remained unchanged. **(C)** Multiple comparisons significance testing showed that for GE, CC, and BC, M1 and F1 did not significantly differ as a pair, nor did M2, F2, M3, and F3. For a given row in a table, cells containing the same color did not significantly differ. A key for grouping is provided.

The modularity algorithm we used could be tuned to identify different sizes and numbers of modules. Increasing the γ parameter produced smaller but more numerous modules (Figure 3A). When we identified two modules for both male and female brain networks, most graph metrics yielded significantly different distributions that paired M1 with F1 and M2 with F2 (Figure 3C). However, introducing a third module did not produce a third distinct network architecture type (Figure 3B). Instead, the results from the two module analysis remained largely unchanged with the original pairings still appearing. The new Male 3 and Female 3 modules consisted of brains with similar GEs, CCs, and BCs to M2 and F2 while significantly differing from M1 and F1 (*p* < 0.05). For both sexes, the third module consisted entirely of brains originating from either M2 or F2. As a result, we concluded there were only two statistically distinct architectures in both male and female brains.

### Architectural grouping was predicted by edge distributions within and across brain regions

**FIGURE 4:**
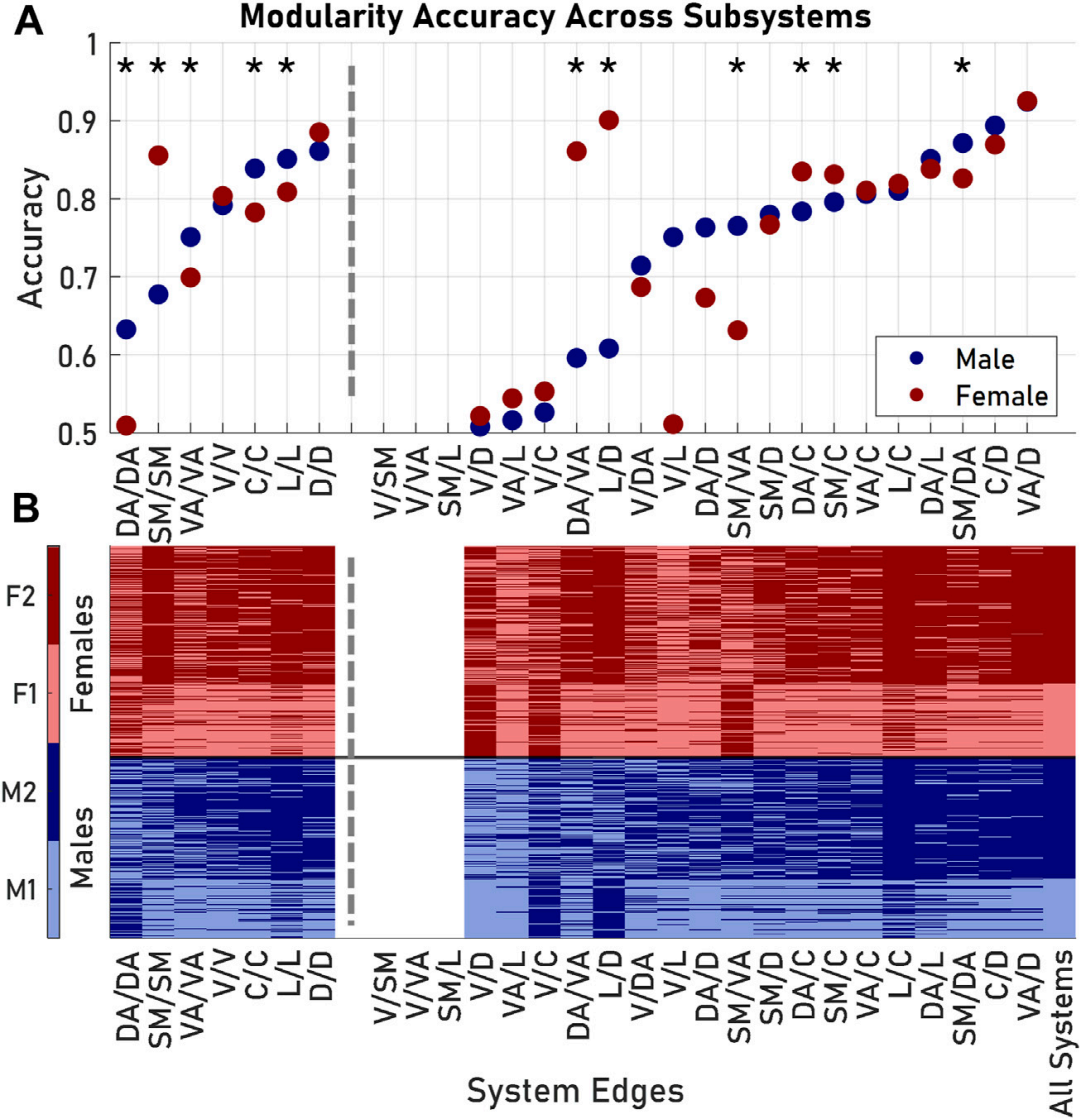
Modularity exhibited nonuniform accuracy when considering different subsets of edges from the original Schaefer 100 parcellation. **(A)** Accuracy, or the fraction of subjects placed in the correct module, was dependent on the subset of nodes included in the similarity matrix formulation. Asterisks indicate subsets where males and females had significantly different accuracies. **(B)** Group identification according to each modularity analysis. Visual/somatomotor, visual/ventral attention, and somatomotor/limbic edges failed to produce comparable modules. Dashed lines delineate between results from edges from one Yeo 7 system (left) and results from edges between two Yeo 7 systems (right). System pairs for each subset listed as System 1/System 2. Abbreviations used: V, Visual; SM, Somatomotor; DA, Dorsal Attention; VA, Ventral Attention; L, Limbic; C, Control; D, Default.

Examining the entire architecture structure across large populations is computationally intensive and requires copious data depending on the resolution of the parcellation used. As an alternative, more efficient approach, we next considered if these architectural groupings in male and female brains could be predicted using only a subset of the SC network *within* a brain subregion. Using the subset of Schaefer 100 edges that correspond to the connections within a single Yeo 7 region, we observed that the distributions of several network features (density, mean nodal strength, and mean clustering coefficient) of these subset matrices recapitulated the significant pairings when considering the whole network (*p* < 0.05). Among these single brain region edges, dorsal-dorsal and limbic-limbic edges performed as the best features to predict overall brain architecture. Edges within these regions were at least 80% accurate in assigning both males and females to their original modules from the whole brain network modularity analysis (Figure 4A).

A second classifier approach would use the prediction of architecture based on edge weights *across* brain regions. In this case, we evaluated the unique Schaefer 100 edges between nodes of two systems in the Yeo 7 parcellation. Among the 21 possible inter-region edge weight measures, the visual-somatomotor, visual-ventral attention, and somatomotor-limbic submatrices failed to produce two modules (Figure 4B). For the remaining 18 inter-region weights, system connections yielded varying degrees of success in classifying brain architecture with this single network feature (accuracy: 0.51–0.92), with the most accurate edges for both sexes coming from the ventral attention and default systems (Figure 4A). In six of the 18 possible inter-region edge weight classifiers (somatomotor-dorsal attention, somatomotor-ventral attention, somatomotor-control, dorsal attention-ventral attention, dorsal attention-control, and limbic-control) there was a significant difference between the accuracy in predicting grouping between male and female brain architectures (*p* < 0.05, Bonferroni corrected). In general, submatrices with high accuracies for males were also accurate for females; the converse was not true. The difference was not significant overall, and the average accuracy (for applicable subsets) for males was 0.713 and the average accuracy for females was 0.732 (*p* = 0.583).

### Transition points in neural dynamics follow architectural groupings

**FIGURE 5:**
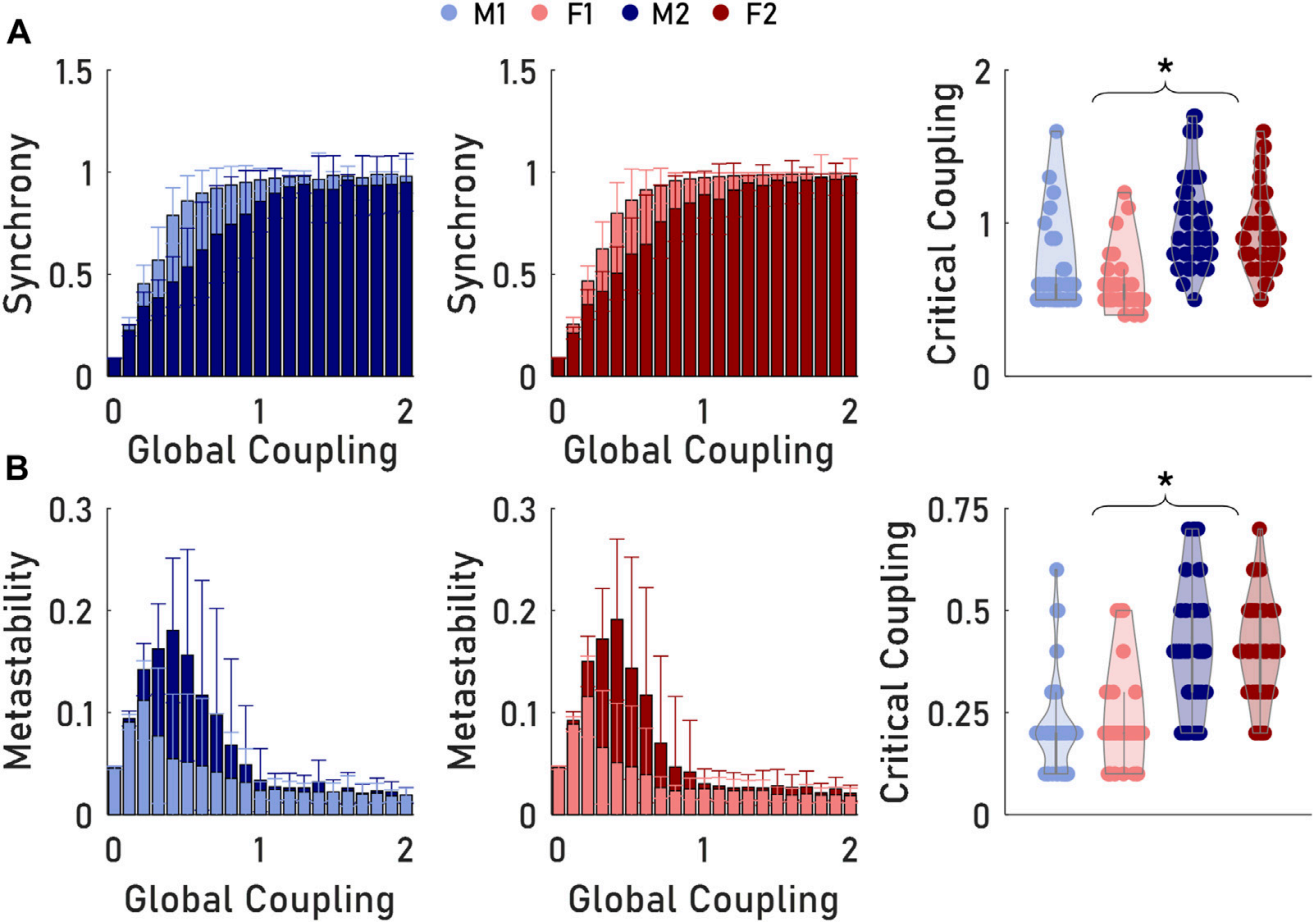
Modules produced distinct patterns of Kuramoto oscillator outputs. **(A)** Synchrony reached its peak at a lower critical coupling strength for M1 and F1 networks than M2 and F2. **(B)** Metastability achieved a greater peak in M2 and F2 modules, but at greater critical coupling values. Critical coupling for synchrony for a given structural connectivity (SC) network was computed as the first global coupling input that yielded synchronization within 10% of the maximum synchrony. Critical coupling for metastability was computed as the global coupling input that corresponded to the Kuramoto oscillator model with the greatest metastability. Asterisks between Group 1 and Group 2 modules indicate that the distributions significantly differed between pairings but not within pairings.

After determining the distinct architectural subgroups in both the male and female population, we next considered if these groupings would also produce a similar separation in Kuramoto oscillator model (KM) predicted neural dynamics. Our analysis of the relationship between synchrony and coupling strength showed a direct architecture-dependence of each model’s ability to cohere its oscillators into unified states (Figure 5A). Comparing respective areas under each subject’s synchrony vs. coupling strength curves, groupings M1 and F1 demonstrated significantly higher synchronies than M2 and F2 for most coupling values before reaching total synchronization (*p* < 0.05). In direct comparison, groupings M1 and F1 were not significantly different in their synchronization over this range of coupling strengths (*p* = 0.768). Likewise, M2 and F2 were not significantly different in synchronization across the coupling range (*p* = 0.789).

We also considered our KMs’ metastability and coupling strength relationships. Over the range of coupling strengths studied, we observed points of maximum metastability for all four distinct brain architectures (Figure 5B). In general, M2 and F2 yielded typically significantly greater metastabilities than M1 and F1. Similar to our synchrony results, the unique male architectures (M1, M2) showed significant differences in their metastability vs. coupling strength curves (*p* < 0.05). Likewise, the female architectures (F1, F2) were also significantly different in their metastability over coupling strength range (*p* < 0.05). No differences were observed when comparing the metastability generated in the M1 and F1 models, nor were differences observed when comparing the M2 and F2 architectures (*p* = 0.902 and *p* = 0.917, respectively). In addition, all networks trended to metastability values of 0 as total synchronization was reached.

With the differences in the synchronization and metastability established across the coupling strength range, we next examined critical coupling strengths to achieve synchrony or peak metastability for all networks in our sample. Synchrony critical coupling for a given network was chosen as the first coupling strength that yielded 90% of that network’s peak synchrony. M1 and F1 (mean $K_{crit,syn}$ = 0.62 and 0.57, respectively) synchronized at significantly lower coupling strengths than M2 and F2 (mean $K_{crit,syn}$ = 1.02 and 0.96, respectively) (*p* < 0.05). However, eventually all modules plateaued to the same maximum synchrony levels at higher coupling levels. Metastability critical coupling for a given network was chosen as the coupling strength that corresponded to the Kuramoto model with the peak metastability. Similar to synchrony critical coupling, metastability reached a critical value at significantly lower coupling strengths in M1 and F1 (mean $K_{crit,mts}$ = 0.21 and 0.21, respectively) than M2 and F2 (mean $K_{crit,mts}$ = 0.42 and 0.41, respectively) (*p* < 0.05). However, the critical coupling strength was not different between M1 and F1 (*p* = 0.998), nor was it different between M2 and F2 (*p* = 0.939).

### Brain architectures exhibited differential response to structural lesions

**FIGURE 6:**
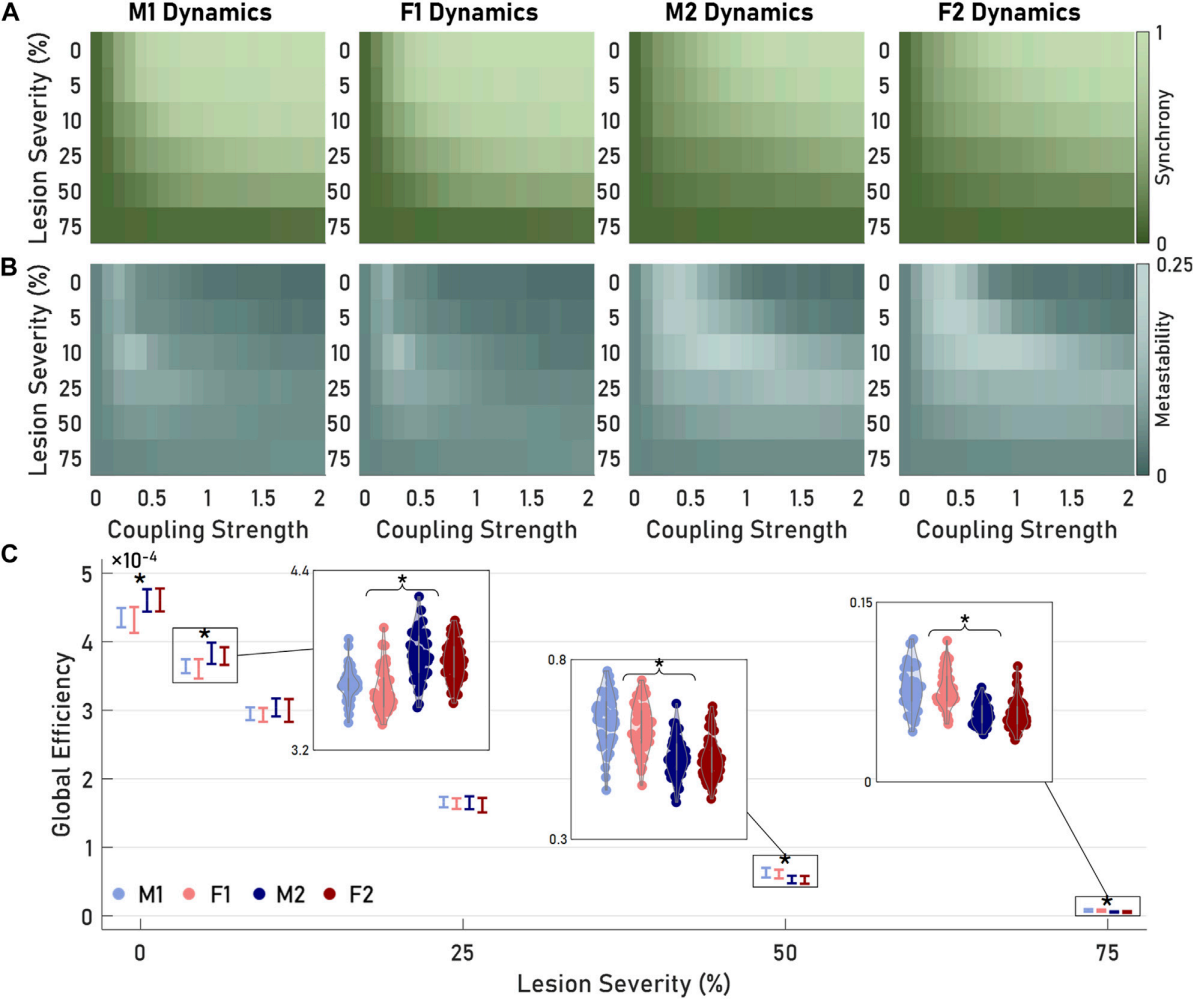
Lesioning altered both the structure and neural dynamics of sampled networks. **(A)** As lesioning increased, synchrony decreased for all coupling strengths in all modules. M1 and F1 maintained greater levels of peak synchronization compared to M2 and F2. **(B)** Max metastability initially increased as result of lesion, but decreased after further lesioning. **(C)** Global efficiency (GE) decreased as a function of lesion severity. Between 10% and 25% lesioning, GE did not exhibit the paired M1-F1 and M2-F2 significance previously seen. Beyond 25% lesioning, M1 and F1 networks exhibited greater GE than M2 and F2. Asterisks denote significant pairings consistent with previous results. Group 1 and Group 2 modules distributions significantly differed between pairings but not within pairings.

At this point in our analysis, our results showed that the unique brain architectures led to differences in neural dynamics which largely followed the original differences in architectures. However, these differences may trace to a small number of specific nodes among architectures or may originate from broader differences in the wiring across the SC groupings. Determining how these differences in dynamics and connectivity emerge among SC subgroups may be particularly important to study the consequences of traumatic injury, which produces a pattern of lesions throughout the brain that depends on the impact location, magnitude, and physical brain size (Donat et al., 2021). Using a modified approach from Alstott et al., 2009, we performed cumulative targeted lesions on our network sample (details regarding cumulative lesions can be found in Supplemental Material S1. We then simulated neural dynamics using KMs on the lesioned networks, again computing synchrony and metastability (Figures 6A,B). For all modules, lesioning impacted model dynamics across a range of coupling strength inputs. Synchrony exhibited a negative relationship with lesioning; not only did KMs converge to lower levels of synchronization, but greater coupling strengths were required to achieve these plateaus (Figure 6A). At the most severe lesion (75% of nodes severed from the network), KMs failed to achieve any meaningful amount of synchronization. The impact on metastability was less direct (Figure 6B). In all modules, a local maximum in metastability appeared between lesions of 5% and 25% and coupling strengths of 0 and 2. As we increased lesion severity, metastability critical coupling mostly increased. The effect on metastability was most pronounced in M2 and F2. Again, the most severe lesions prevented Kuramoto oscillators from being able to coalesce into states, thus minimizing metastability across all coupling strengths.

Despite targeting the same nodes to lesion across all sampled networks, our simulated injuries produced effects on the SC networks which differed significantly among modules. Every network exhibited decreased GE with increased lesioning (Figure 6C). However, SC networks from groupings M2 and F2 demonstrated significantly greater decrease in GE than those from M1 and F1 (mean change in GE from all lesions for M1, F1, M2, and F2, respectively: 2.56 × 10^−4^, 2.55 × 10^−4^, 2.78 × 10^−4^, 2.81 × 10^−4^; *p* < 0.05). When examining the resulting lesioned SC, our sampled networks recapitulated the trends from the entire dataset with the mean M1 and F1 GEs appearing significantly lower than the mean GEs of M2 and F2 at the least severe lesions (Figure 6B; mean $GE_{5\%}$ for M1, F1, M2, and F2, respectively: 3.64 × 10^−4^, 3.60 × 10^−4^, 3.83 × 10^−4^, 3.79 × 10^−4^; *p* < 0.05). When we increased lesion severity, these differences in lesioned SCs disappeared between 10% and 25% injury (mean $GE_{25\%}$ for M1, F1, M2, and F2, respectively: 1.66 × 10^−4^, 1.64 × 10^−4^, 1.65 × 10^−4^, 1.62 × 10^−4^; *p* > 0.05 for all comparisons). At the most severe injury level, M1 and F1 networks had significantly greater GEs than M2 and F2 (mean $GE_{75\%}$ for M1, F1, M2, and F2, respectively: 0.0798 × 10^−4^, 0.0770 × 10^−4^, 0.0566 × 10^−4^, 0.0567 × 10^−4^; *p* > 0.05).

## Discussion

We showed that a large population of human brain architectures can be distilled into two distinctly shaped network representations for both male and female brains. The groupings converged further, as we found the unique architectures for male and female brains did not significantly differ across sex; two structural networks captured accurately the entire population of human brains analyzed. Despite high similarity metrics between all brains in our population (respective intramodule and intermodule mean correlations of 0.942 and 0.906) we showed that this slight difference was significant enough to produce distinct distributions of structural graph metrics, simulated neural dynamics, and responses to lesioning. For modest lesions to the network, one architectural grouping demonstrated more impairment in neural dynamics than the other architecture. At the highest injury levels, the differences in dynamics between these two architectural groupings disappeared. Together, these results suggested that brain architecture and the response to traumatic brain injury (TBI) may be interrelated, raising the possibility that brain architecture type could be an important factor in predicting the consequences of head impact in humans.

The methods we used in this study were derived from several previous articles and attempted to address methodological limitations common to these techniques. Our KM methodology was largely inspired by Cabral et al., 2011., who used KMs in conjunction with the Balloon-Windkessel (BW) model to simulate functional connectivity, optimizing the model parameters to match the measured functional connectivity in a human subject. One difference in our approach was using a model that did not include signal latency among brain areas, an approximation that allowed us to model the dynamics across a far larger number of architectures than previously examined by Cabral or others (Petkoski and Jirsa, 2019; Wu et al., 2022a). Such a method more closely matched the KM from Allegra Mascaro et al., 2020 that assumed instantaneous coupling between oscillators albeit at low frequencies. Moreover, we focused on how the coupled neural dynamics—and not simulated function—would change across this range of architectures and allowed us to determine whether groupings of dynamics would change with the coupling strength (they did not). We were encouraged that these approximations still provided a reasonable fidelity to trends found within the original study (Cabral et al., 2011), where synchrony plateaued as a function of coupling strength and that metastability peaked at intermediate coupling strengths. Interestingly, lesioning had comparable effects to increasing delay. Oscillators became less synchronous and metastability increased perhaps because removing nodes reduced the model’s ability to coordinate oscillations.

A second limitation was employing targeted lesioning rather than trying to model explicitly the lesion patterns that occur in TBI. The lesioning approach was modeled after Alstott et al., 2009, who ranked and lesioned nodes in a single network according to various SC metrics including centrality and degree. Like our approach, Alstott et al. systematically increased lesion severity and evaluated the impact of each lesion on the resulting network features. To produce an analogous injury while maintaining consistency across our set of 1,065 networks, we used the mean degree ranking. Although this lesioning methodology was sufficient to study injuries, especially from a topological perspective, it did not represent the true distribution of lesions that occur during a TBI (Kraft et al., 2012; Donat et al., 2021). The pattern of damage after each TBI is unique and depends on the circumstances describing the impact (Gabler et al., 2016, 2018). Although we could use a method incorporating brain biomechanics and strain injury criteria to produce a more realistic injury pattern (Kraft et al., 2012), this approach would make the process of identifying critical nodes more difficult.

It should be noted that lesioning can be used to model the effects of other forms of neurodegeneration. Allegra Mascaro et al., 2020, used a KM to examine how lesions from stroke altered network communications. Others have similarly examined the network disruptions associated with epilepsy (Olmi et al., 2019). Brain disorders associated with axonal deficits, such as amyotrophic lateral sclerosis, Alzheimer’s, or multiple sclerosis can be represented by similarly altering the edges in a SC network (Fornito et al., 2015). Together, along with TBI, these conditions all represent disruptions to the axonal connections between different brain regions. Aerts et al., 2016 go on to describe how such disruptions intersect very well with graph theory, and network metrics can be leveraged to demonstrate the effects of lesions from disease or injury. The conclusions from this study regarding architecture-dependence of lesions’ effects may share some applicability and relevance to these other neurodegenerative conditions.

### Contextualizing brain architecture typing research

In addition to the specific comparisons to Cabral et al., 2011 and Alstott et al., 2009, as well as the other cases in which network lesions may be representative of neurodegeneration, we would like to draw attention to other studies in the field of identifying “types” of brains. Our study’s impact comes from its distinctions here and the potential applications of our brain typing findings. Attempting to characterize brain “types” has been explored by other researchers; our primary contribution to this field was using networks to determine whether there is an architecture-dependent vulnerability among these types. Other methods include analyzing the distribution of grey matter volumes or pre-dividing networks based on demographics and analyzing the effect of these subgroups (Ingalhalikar et al., 2014; Joel et al., 2015, 2018; Tyan et al., 2017). As a result, the methods of these studies are limited in their applicability to understanding how brain function is related to white matter structure. For modeling the consequences of head impact, methods that focus on grey matter distribution may help create individual-specific models where the distribution and properties of grey matter may lead to changes in injury risk across subjects (Giordano et al., 2017). Conversely, studies that pre-divide according to sex may capture general trends that exist between male and female subjects but fail to define more nuanced trends in the population. Other past work considered how the size and shape of the brain can affect the relative susceptibility of the brain to impact and offered new insight for scaling impact magnitude to account for risk across different populations (Wu et al., 2020). However, none of these efforts incorporated a simultaneous analysis of the network structure and its separate role in risk prediction.

### Architecture-dependent consequences of lesions

Our ability to identify a brain’s architecture type did not require consideration of the entire brain network. Instead, with relatively high accuracy, we could choose subsets of the network features and accurately recapture our original architecture groupings. Conversely, other nodal subsets do not show any meaningful difference across the population. Both outcomes are reminiscent of algorithms to determine edges and edge weight distributions in both structural and functional networks which successfully separate healthy controls from individuals with schizophrenia, Alzheimer’s disease, and depression (Dai et al., 2012; Chi et al., 2015; Gutiérrez-Gómez et al., 2020). At an individual level, generative models produce structural connectome wiring rules for a given network state (Betzel and Bassett, 2017) to represent the probability of an edge existing within a network of interest, such as one from a schizophrenic patient (Zhang et al., 2021). In other cases, small patterns of connections, or motifs, have been used to identify disease states in brain networks (Sporns and Kötter, 2004; Friedman et al., 2015). Rather than disease in our case, we instead identified network types that exhibited distinct edge probabilities and arrangements. Subsets of nodes could act as biomarkers to classify a network into an architecture type.

### Different architecture types exhibit different levels of susceptibility to injury

There is a consensus that the impact magnitude and duration is correlated to brain injury outcome (el Sayed et al., 2008; Meaney et al., 2014; Gabler et al., 2016; Alshareef et al., 2020). Numerous injury risk functions have been produced linking increasing kinematics and deformations within the brain to TBI likelihood (Funk et al., 2007; Rowson et al., 2012; Sanchez et al., 2017; Gabler et al., 2018). Two past studies suggested that incorporating features of the brain network can explain why different injury outcomes may occur in similar impact conditions (Aerts et al., 2016; Anderson et al., 2020). We extended these analyses one step further to consider how the consequences of injury across a population may arise from differences in the architectures among subjects. Indeed, it is known that populations are not homogenous enough to assume a general response to TBI (Broglio et al., 2017). However, if one needed to calculate the individual risk for each individual architecture, the computational burden would be overwhelming (Raizman et al., 2020). Here, our ability to coalesce many individual architectures into two distinct groups for male and female brains and, further, into two overarching architecture types, makes the estimation of individual risk more tenable. Moreover, the ability to designate a brain architecture type based on the connectivity characteristics within a specific brain subregion makes it possible to quickly identify, sort, and prospectively determine relative risk for any individual brain in future studies.

One key result was that our differences among architectures disappeared after a moderate number of areas were injured. These findings implied that architecture-dependent changes only applied for mild injury levels, especially if the injury pattern was focused on nodes with relatively high degree. Once the extent of injury exceeded roughly half of the network, the networks failed to produce any meaningful level of synchronization, produced low metastability, and did not show any significant differences in these changes across architecture type. However, even at the more severe lesion levels, the global efficiency measures showed the remaining network structure still retained some differentiable capacity to transmit information through the network, albeit at a negligibly inefficient level. Lesions at this severity exceeded any reasonable expectations of the injuries typically observed in these types of analyses. Instead, the domain of injury that is of interest remains mild TBI (concussion) which usually occurs when approximately 10–30% of brain tissue exceeds injurious strain thresholds (Wu et al., 2021). It is here that brain types can be used to further understand the heterogenous nature of TBI.

## Conclusion

Our work highlights the possibility that the consequences of an impact to the brain may depend on the initial brain architecture. On its own, this work indicates that brain network architecture should be considered when attempting to predict the injury risk after an impact. One likely consequence is that different types of architectures may make some brains more vulnerable to impact in specific directions, while other architectures could be vulnerable to injury from impacts in a different direction. This potential to generate a customized vulnerability for each architecture points to the possibility of designing head protective equipment that is either customized to one architecture or adequately protects all architectures equally. In the future, these results imply that knowing a person’s architecture type ahead of time can enable him/her to take precautions against impacts that will be particularly injurious.

## Data Availability

Publicly available datasets were analyzed in this study. This data can be found here: The diffusion MRI and functional MRI data are available through Human Connectome Project (https://www.humanconnectome.org/study/hcp-young-adult). The parcellation atlas is publicly available on Github (https://github.com/ThomasYeoLab/CBIG/tree/master/stable_projects/brain_parcellation/Schaefer2018_LocalGlobal). Additional scripts and data used by the authors can be found at https://itsja.red/KuOs.

## References

[B1] Aerts H. Fias W. Caeyenberghs K. Marinazzo D. (2016). Brain networks under attack: Robustness properties and the impact of lesions. Brain 139, 3063–3083. 10.1093/brain/aww194 27497487

[B2] Allegra Mascaro A. L. Falotico E. Petkoski S. Pasquini M. Vannucci L. Tort-Colet N. (2020). Experimental and computational study on motor control and recovery after stroke: Toward a constructive loop between experimental and virtual embodied neuroscience. Front. Syst. Neurosci. 14, 31. 10.3389/fnsys.2020.00031 32733210 PMC7359878

[B3] Alshareef A. Giudice J. S. Forman J. Shedd D. F. Reynier K. A. Wu T. (2020). Biomechanics of the human brain during dynamic rotation of the head. J. Neurotrauma 37, 1546–1555. 10.1089/neu.2019.6847 31952465 PMC7307677

[B4] Alstott J. Breakspear M. Hagmann P. Cammoun L. Sporns O. (2009). Modeling the impact of lesions in the human brain. PLoS Comput. Biol. 5, e1000408. 10.1371/journal.pcbi.1000408 19521503 PMC2688028

[B5] Anderson E. D. Giudice J. S. Wu T. Panzer M. B. Meaney D. F. (2020). Predicting concussion outcome by integrating finite element modeling and network analysis. Front. Bioeng. Biotechnol. 8, 309. 10.3389/fbioe.2020.00309 32351948 PMC7174699

[B6] Asemota A. O. George B. P. Bowman S. M. Haider A. H. Schneider E. B. (2013). Causes and trends in traumatic brain injury for United States adolescents. J. Neurotrauma 30, 67–75. 10.1089/neu.2012.2605 22989254

[B7] Bain A. C. Meaney D. F. (2000). Tissue-level thresholds for axonal damage in an experimental model of central nervous system white matter injury. J. Biomech. Eng. 122, 615–622. 10.1115/1.1324667 11192383

[B8] Bassett D. S. Sporns O. (2017). Network neuroscience. Nat. Neurosci. 20, 353–364. 10.1038/nn.4502 28230844 PMC5485642

[B9] Benice T. S. Rizk A. Kohama S. Pfankuch T. Raber J. (2006). Sex-differences in age-related cognitive decline in C57BL/6J mice associated with increased brain microtubule-associated protein 2 and synaptophysin immunoreactivity. Neuroscience 137, 413–423. 10.1016/j.neuroscience.2005.08.029 16330151

[B10] Betzel R. F. Bassett D. S. (2017). Multi-scale brain networks. Neuroimage 160, 73–83. 10.1016/j.neuroimage.2016.11.006 27845257 PMC5695236

[B11] Bretzin A. C. Covassin T. Wiebe D. J. Stewart W. (2021). Association of sex with adolescent soccer concussion incidence and characteristics. JAMA Netw. Open 4, e218191. 10.1001/jamanetworkopen.2021.8191 33904911 PMC8080231

[B12] Broglio S. P. Lapointe A. O’Connor K. L. McCrea M. (2017). Head impact density: A model to explain the elusive concussion threshold. J. Neurotrauma 34, 2675–2683. 10.1089/neu.2016.4767 28381134 PMC5647505

[B13] Cabral J. Hugues E. Sporns O. Deco G. (2011). Role of local network oscillations in resting-state functional connectivity. Neuroimage 57, 130–139. 10.1016/j.neuroimage.2011.04.010 21511044

[B14] Caeyenberghs K. Leemans A. Leunissen I. Gooijers J. Michiels K. Sunaert S. (2014). Altered structural networks and executive deficits in traumatic brain injury patients. Brain Struct. Funct. 219, 193–209. 10.1007/s00429-012-0494-2 23232826

[B15] Chi M. Guo S. Ning Y. Li J. Qi H. Gao M. (2015). Discriminative analysis of major depressive disorder and anxious depression using support vector machine. J. Comput. Theor. Nanosci. 12, 1395–1401. 10.1166/jctn.2015.3903

[B16] Córdova-Palomera A. Kaufmann T. Persson K. Alnæs D. Doan N. T. Moberget T. (2017). Disrupted global metastability and static and dynamic brain connectivity across individuals in the Alzheimer’s disease continuum. Sci. Rep. 7, 40268. 10.1038/srep40268 28074926 PMC5225495

[B17] Cumin D. Unsworth C. P. (2007). Generalising the Kuramoto model for the study of neuronal synchronisation in the brain. Phys. D. Nonlinear Phenom. 226, 181–196. 10.1016/j.physd.2006.12.004

[B18] Dai Z. Yan C. Wang Z. Wang J. Xia M. Li K. (2012). Discriminative analysis of early Alzheimer’s disease using multi-modal imaging and multi-level characterization with multi-classifier (M3). Neuroimage 59, 2187–2195. 10.1016/j.neuroimage.2011.10.003 22008370

[B19] Dall’Acqua P. Johannes S. Mica L. Simmen H.-P. Glaab R. Fandino J. (2017). Functional and structural network recovery after mild traumatic brain injury: A 1-year longitudinal study. Front. Hum. Neurosci. 11, 280. 10.3389/fnhum.2017.00280 28611614 PMC5447750

[B20] Dollé J.-P. Jaye A. Anderson S. A. Ahmadzadeh H. Shenoy V. B. Smith D. H. (2018). Newfound sex differences in axonal structure underlie differential outcomes from *in vitro* traumatic axonal injury. Exp. Neurol. 300, 121–134. 10.1016/j.expneurol.2017.11.001 29104114 PMC6495524

[B21] Domingos P. (2012). A few useful things to know about machine learning. Commun. ACM 55, 78–87. 10.1145/2347736.2347755

[B22] Donat C. K. Yanez Lopez M. Sastre M. Baxan N. Goldfinger M. Seeamber R. (2021). From biomechanics to pathology: Predicting axonal injury from patterns of strain after traumatic brain injury. Brain 144, 70–91. 10.1093/brain/awaa336 33454735 PMC7990483

[B23] Dymek M. Ptak M. Fernandes F. A. O. (2021). Design and virtual testing of American football helmets–A review. Arch. Comput. Methods Eng. 29, 1277–1289. 10.1007/s11831-021-09621-7

[B24] el Sayed T. Mota A. Fraternali F. Ortiz M. (2008). Biomechanics of traumatic brain injury. Comput. Methods Appl. Mech. Eng. 197. 10.1016/j.cma.2008.06.006

[B25] Fanton M. Sganga J. Camarillo D. B. (2019). Vulnerable locations on the head to brain injury and implications for helmet design. J. Biomech. Eng. 141. 10.1115/1.4044876 31523753

[B26] Fornito A. Zalesky A. Breakspear M. (2015). The connectomics of brain disorders. Nat. Rev. Neurosci. 16, 159–172. 10.1038/nrn3901 25697159

[B27] Fredriksson R. Shin J. Untaroiu C. D. (2011). Potential of pedestrian protection systems—a parameter study using finite element models of pedestrian dummy and generic passenger vehicles. Traffic Inj. Prev. 12, 398–411. 10.1080/15389588.2011.566655 21823948

[B28] Friedman E. J. Young K. Tremper G. Liang J. Landsberg A. S. Schuff N. (2015). Directed network motifs in Alzheimer’s disease and mild cognitive impairment. PLOS ONE 10, e0124453. 10.1371/journal.pone.0124453 25879535 PMC4400037

[B29] Fukushima M. Sporns O. (2018). Comparison of fluctuations in global network topology of modeled and empirical brain functional connectivity. PLoS Comput. Biol. 14, e1006497. 10.1371/journal.pcbi.1006497 30252835 PMC6173440

[B30] Funk J. R. Duma S. M. Manoogian S. J. Rowson S. (2007). Biomechanical risk estimates for mild traumatic brain injury. Annu. Proc. Assoc. Adv. Automot. Med. 51, 343–361. 18184501 PMC3217524

[B31] Gabler L. F. Crandall J. R. Panzer M. B. (2016). Assessment of kinematic brain injury metrics for predicting strain responses in diverse automotive impact conditions. Ann. Biomed. Eng. 44, 3705–3718. 10.1007/s10439-016-1697-0 27436295

[B32] Gabler L. F. Crandall J. R. Panzer M. B. (2018). Development of a metric for predicting brain strain responses using head kinematics. Ann. Biomed. Eng. 46, 972–985. 10.1007/s10439-018-2015-9 29594689

[B33] Gilbert N. Bernier R. A. Calhoun V. D. Brenner E. Grossner E. Rajtmajer S. M. (2018). Diminished neural network dynamics after moderate and severe traumatic brain injury. PLOS ONE 13, e0197419. 10.1371/journal.pone.0197419 29883447 PMC5993261

[B34] Giordano C. Zappalà S. Kleiven S. (2017). Anisotropic finite element models for brain injury prediction: The sensitivity of axonal strain to white matter tract inter-subject variability. Biomech. Model. Mechanobiol. 16, 1269–1293. 10.1007/s10237-017-0887-5 28233136 PMC5511602

[B35] Giudice J. S. Alshareef A. Wu T. Gancayco C. A. Reynier K. A. Tustison N. J. (2020a). An image registration-based morphing technique for generating subject-specific brain finite element models. Ann. Biomed. Eng. 48, 2412–2424. 10.1007/s10439-020-02584-z 32725547

[B36] Giudice J. S. Caudillo A. Mukherjee S. Kong K. Park G. Kent R. (2020b). Finite element model of a deformable American football helmet under impact. Ann. Biomed. Eng. 48, 1524–1539. 10.1007/s10439-020-02472-6 32034610

[B37] Gutiérrez-Gómez L. Vohryzek J. Chiêm B. Baumann P. S. Conus P. Cuenod K. D. (2020). Stable biomarker identification for predicting schizophrenia in the human connectome. NeuroImage Clin. 27, 102316. 10.1016/j.nicl.2020.102316 32623137 PMC7334612

[B38] Hajiaghamemar M. Wu T. Panzer M. B. Margulies S. S. (2020). Embedded axonal fiber tracts improve finite element model predictions of traumatic brain injury. Biomech. Model. Mechanobiol. 19, 1109–1130. 10.1007/s10237-019-01273-8 31811417 PMC7203590

[B39] Hellyer P. J. Scott G. Shanahan M. Sharp D. J. Leech R. (2015). Cognitive flexibility through metastable neural dynamics is disrupted by damage to the structural connectome. J. Neurosci. 35, 9050–9063. 10.1523/JNEUROSCI.4648-14.2015 26085630 PMC4469735

[B40] Hodgkin A. L. Huxley A. F. (1952). A quantitative description of membrane current and its application to conduction and excitation in nerve. J. Physiology 117, 500–544. 10.1113/jphysiol.1952.sp004764 PMC1392413 12991237

[B41] Honey C. J. Sporns O. (2008). Dynamical consequences of lesions in cortical networks. Hum. Brain Mapp. 29, 802–809. 10.1002/hbm.20579 18438885 PMC6870962

[B42] Ingalhalikar M. Smith A. Parker D. Satterthwaite T. D. Elliott M. A. Ruparel K. (2014). Sex differences in the structural connectome of the human brain. Proc. Natl. Acad. Sci. U. S. A. 111, 823–828. 10.1073/pnas.1316909110 24297904 PMC3896179

[B43] Izhikevich E. M. (2003). Simple model of spiking neurons. IEEE Trans. Neural Netw. 14, 1569–1572. 10.1109/TNN.2003.820440 18244602

[B44] Joel D. Berman Z. Tavor I. Wexler N. Gaber O. Stein Y. (2015). Sex beyond the genitalia: The human brain mosaic. Proc. Natl. Acad. Sci. U. S. A. 112, 15468–15473. 10.1073/pnas.1509654112 26621705 PMC4687544

[B45] Joel D. Persico A. Salhov M. Berman Z. Oligschläger S. Meilijson I. (2018). Analysis of human brain structure reveals that the brain “types” typical of males are also typical of females, and vice versa. Front. Hum. Neurosci. 12, 399. 10.3389/fnhum.2018.00399 30405373 PMC6204758

[B46] Kleiven S. von Holst H. (2002). Consequences of head size following trauma to the human head. J. Biomechanics 35, 153–160. 10.1016/S0021-9290(01)00202-0 11784533

[B47] Kraft R. H. Mckee P. J. Dagro A. M. Grafton S. T. (2012). Combining the finite element method with structural connectome-based analysis for modeling neurotrauma: Connectome neurotrauma mechanics. PLoS Comput. Biol. 8, e1002619. 10.1371/journal.pcbi.1002619 22915997 PMC3420926

[B48] Kurt M. Laksari K. Kuo C. Grant G. A. Camarillo D. B. (2017). Modeling and optimization of airbag helmets for preventing head injuries in bicycling. Ann. Biomed. Eng. 45, 1148–1160. 10.1007/s10439-016-1732-1 27679447

[B49] Lee W. H. Bullmore E. Frangou S. (2017). Quantitative evaluation of simulated functional brain networks in graph theoretical analysis. Neuroimage 146, 724–733. 10.1016/j.neuroimage.2016.08.050 27568060 PMC5312789

[B50] Mao H. Zhang L. Jiang B. Genthikatti V. V. Jin X. Zhu F. (2013). Development of a finite element human head model partially validated with thirty five experimental cases. J. Biomech. Eng. 135, 111002. 10.1115/1.4025101 24065136

[B51] Meaney D. F. Morrison B. Dale Bass C. (2014). The mechanics of traumatic brain injury: A review of what we know and what we need to know for reducing its societal burden. J. Biomech. Eng. 136, 021008. 10.1115/1.4026364 24384610 PMC4023660

[B52] Messé A. Rudrauf D. Giron A. Marrelec G. (2015). Predicting functional connectivity from structural connectivity via computational models using MRI: An extensive comparison study. Neuroimage 111, 65–75. 10.1016/j.neuroimage.2015.02.001 25682944

[B53] Miller G. F. DePadilla L. Xu L. (2021). Costs of nonfatal traumatic brain injury in the United States, 2016. Med. Care 59, 451–455. 10.1097/MLR.0000000000001511 33528230 PMC8026675

[B54] Newman M. E. J. (2006). Modularity and community structure in networks. Proc. Natl. Acad. Sci. U. S. A. 103, 8577–8582. 10.1073/pnas.0601602103 16723398 PMC1482622

[B55] Olmi S. Petkoski S. Guye M. Bartolomei F. Jirsa V. (2019). Controlling seizure propagation in large-scale brain networks. PLoS Comput. Biol. 15, e1006805. 10.1371/journal.pcbi.1006805 30802239 PMC6405161

[B56] Petkoski S. Jirsa V. K. (2019). Transmission time delays organize the brain network synchronization. Phil. Trans. R. Soc. A 377, 20180132. 10.1098/rsta.2018.0132 31329065 PMC6661323

[B57] Raizman R. Tavor I. Biegon A. Harnof S. Hoffmann C. Tsarfaty G. (2020). Traumatic brain injury severity in a network perspective: A diffusion MRI based connectome study. Sci. Rep. 10, 9121. 10.1038/s41598-020-65948-4 32499553 PMC7272462

[B58] Reichardt J. Bornholdt S. (2006). Statistical mechanics of community detection. Phys. Rev. E 74, 016110. 10.1103/PhysRevE.74.016110 16907154

[B59] Reynier K. Giudice J. Forman J. Panzer M. (2021). “Preliminary investigation of sex-specific geometries and head kinematics on brain response using finite element brain models in automotive crash loading conditions,” in International research council on biomechanics of injury, 357–358.

[B60] Rowson S. Duma S. M. Beckwith J. G. Chu J. J. Greenwald R. M. Crisco J. J. (2012). Rotational head kinematics in football impacts: An injury risk function for concussion. Ann. Biomed. Eng. 40, 1–13. 10.1007/s10439-011-0392-4 22012081 PMC10465647

[B61] Rubinov M. Sporns O. (2010). Complex network measures of brain connectivity: Uses and interpretations. Neuroimage 52, 1059–1069. 10.1016/j.neuroimage.2009.10.003 19819337

[B62] Sanchez E. J. Gabler L. F. McGhee J. S. Olszko A. V. Chancey V. C. Crandall J. R. (2017). Evaluation of head and brain injury risk functions using sub-injurious human volunteer data. J. Neurotrauma 34, 2410–2424. 10.1089/neu.2016.4681 28358277

[B63] Schaefer A. Kong R. Gordon E. M. Laumann T. O. Zuo X.-N. Holmes A. J. (2018). Local-global parcellation of the human cerebral cortex from intrinsic functional connectivity MRI. Cereb. Cortex 28, 3095–3114. 10.1093/cercor/bhx179 28981612 PMC6095216

[B64] Schmidt R. LaFleur K. J. R. de Reus M. A. van den Berg L. H. van den Heuvel M. P. (2015). Kuramoto model simulation of neural hubs and dynamic synchrony in the human cerebral connectome. BMC Neurosci. 16, 54–13. 10.1186/s12868-015-0193-z 26329640 PMC4556019

[B65] Sharp D. J. Scott G. Leech R. (2014). Network dysfunction after traumatic brain injury. Nat. Rev. Neurol. 10, 156–166. 10.1038/nrneurol.2014.15 24514870

[B66] Siettos C. Starke J. (2016). Multiscale modeling of brain dynamics: From single neurons and networks to mathematical tools. WIREs Mech. Dis. 8, 438–458. 10.1002/wsbm.1348 27340949

[B67] Sporns O. Kötter R. (2004). Motifs in brain networks. PLoS Biol. 2, e369. 10.1371/journal.pbio.0020369 15510229 PMC524253

[B68] Sullivan S. Eucker S. A. Gabrieli D. Bradfield C. Coats B. Maltese M. R. (2015). White matter tract-oriented deformation predicts traumatic axonal brain injury and reveals rotational direction-specific vulnerabilities. Biomech. Model. Mechanobiol. 14, 877–896. 10.1007/s10237-014-0643-z 25547650 PMC4486640

[B69] Taylor C. A. Bell J. M. Breiding M. J. Xu L. (2017). Traumatic brain injury–related emergency department visits, hospitalizations, and deaths — United States, 2007 and 2013. MMWR. Surveill. Summ. 66, 1–16. Surveillance Summaries. 10.15585/mmwr.ss6609a1 PMC5829835 28301451

[B70] Tyan Y.-S. Liao J.-R. Shen C.-Y. Lin Y.-C. Weng J.-C. (2017). Gender differences in the structural connectome of the teenage brain revealed by generalized q-sampling MRI. NeuroImage Clin. 15, 376–382. 10.1016/j.nicl.2017.05.014 28580294 PMC5447512

[B71] van der Horn H. J. Kok J. G. de Koning M. E. Scheenen M. E. Leemans A. Spikman J. M. (2017). Altered wiring of the human structural connectome in adults with mild traumatic brain injury. J. Neurotrauma 34, 1035–1044. 10.1089/neu.2016.4659 27627836

[B72] van Essen D. C. Smith S. M. Barch D. M. Behrens T. E. J. Yacoub E. Ugurbil K. (2013). The Wu-minn human connectome project: an overview. Neuroimage 80, 62–79. 10.1016/j.neuroimage.2013.05.041 23684880 PMC3724347

[B73] van Pelt K. L. Allred D. Cameron K. L. Campbell D. E. D’Lauro C. J. He X. (2019). A cohort study to identify and evaluate concussion risk factors across multiple injury settings: Findings from the CARE Consortium. Inj. Epidemiol. 6, 1. 10.1186/s40621-018-0178-3 30637568 PMC6330552

[B74] Váša F. Shanahan M. Hellyer P. J. Scott G. Cabral J. Leech R. (2015). Effects of lesions on synchrony and metastability in cortical networks. Neuroimage 118, 456–467. 10.1016/j.neuroimage.2015.05.042 26049146

[B75] Wright R. M. Ramesh K. T. (2012). An axonal strain injury criterion for traumatic brain injury. Biomech. Model. Mechanobiol. 11, 245–260. 10.1007/s10237-011-0307-1 21476072

[B76] Wu T. Alshareef A. Giudice J. S. Panzer M. B. (2019). Explicit modeling of white matter axonal fiber tracts in a finite element brain model. Ann. Biomed. Eng. 47, 1908–1922. 10.1007/s10439-019-02239-8 30877404

[B77] Wu T. Antona-Makoshi J. Alshareef A. Giudice J. S. Panzer M. B. (2020). Investigation of cross-species scaling methods for traumatic brain injury using finite element analysis. J. Neurotrauma 37, 410–422. 10.1089/neu.2019.6576 31382861

[B78] Wu T. Hajiaghamemar M. Giudice J. S. Alshareef A. Margulies S. S. Panzer M. B. (2021). Evaluation of tissue-level brain injury metrics using species-specific simulations. J. Neurotrauma 38, 1879–1888. 10.1089/neu.2020.7445 33446011 PMC8219195

[B79] Wu T. Rifkin J. A. Rayfield A. Panzer M. B. Meaney D. F. (2022a). An interdisciplinary computational model for predicting traumatic brain injury: Linking biomechanics and functional neural networks. Neuroimage 251, 119002. 10.1016/j.neuroimage.2022.119002 35176490

[B80] Wu T. Sato F. Antona-Makoshi J. Gabler L. F. Giudice J. S. Alshareef A. (2022b). Integrating human and nonhuman primate data to estimate human tolerances for traumatic brain injury. J. Biomech. Eng. 144, 071003. 10.1115/1.4053209 34897386

[B81] Xin J. Zhang Y. Tang Y. Yang Y. (2019). Brain differences between men and women: Evidence from deep learning. Front. Neurosci. 13, 185. 10.3389/fnins.2019.00185 30906246 PMC6418873

[B82] Yeh F.-C. Tseng W.-Y. I. (2011). NTU-90: A high angular resolution brain atlas constructed by q-space diffeomorphic reconstruction. Neuroimage 58, 91–99. 10.1016/j.neuroimage.2011.06.021 21704171

[B83] Yeo B. T. T. Krienen F. M. Sepulcre J. Sabuncu M. R. Lashkari D. Hollinshead M. (2011). The organization of the human cerebral cortex estimated by intrinsic functional connectivity. J. Neurophysiol. 106, 1125–1165. 10.1152/jn.00338.2011 21653723 PMC3174820

[B84] Yuan W. Wade S. L. Babcock L. (2015). Structural connectivity abnormality in children with acute mild traumatic brain injury using graph theoretical analysis. Hum. Brain Mapp. 36, 779–792. 10.1002/hbm.22664 25363671 PMC5500248

[B85] Zhang X. Braun U. Harneit A. Zang Z. Geiger L. S. Betzel R. F. (2021). Generative network models of altered structural brain connectivity in schizophrenia. Neuroimage 225, 117510. 10.1016/j.neuroimage.2020.117510 33160087

