# Supplementary material for: Brain architecture-based vulnerability to traumatic injury
Source: Front Bioeng Biotechnol. 2022 Aug 24;10:936082. doi: 10.3389/fbioe.2022.936082 (PMC9448929; doi:10.3389/fbioe.2022.936082)
Supplement: Supplementary file 1 [file DataSheet1.docx]

Supplemental Figures

**Supplemental Figure 1. Nodal rankings used to determine targeted lesion order.** For each network in our sample, we ranked nodes according to degree, eigenvector centrality, and betweenness centrality. We then computed each node’s mean ranking across all networks and sorted those mean rankings. Degree and eigenvector centrality exhibited wider ranges of rankings, indicating that nodes more consistently arranged themselves in the same orders than betweenness centrality. We chose degree to use for our targeted lesioning due to this consistency.

**Supplemental Figure 2. Subsystem graph metric distributions and tables.** For each subset of edges connecting Schaefer 100 nodes belong to either two distinct Yeo 7 systems or within one system we computed mean shortest path length (MSPL), global efficiency (GE), mean clustering coefficient (CC), mean betweenness centrality (BC), density, and mean strength. After performing multiple comparisons corrections on the distributions for Male 1 (M1), Male 2 (M2), Female 1 (F1), and Female 2 (F2) we report their respective means plus/minus one standard error.


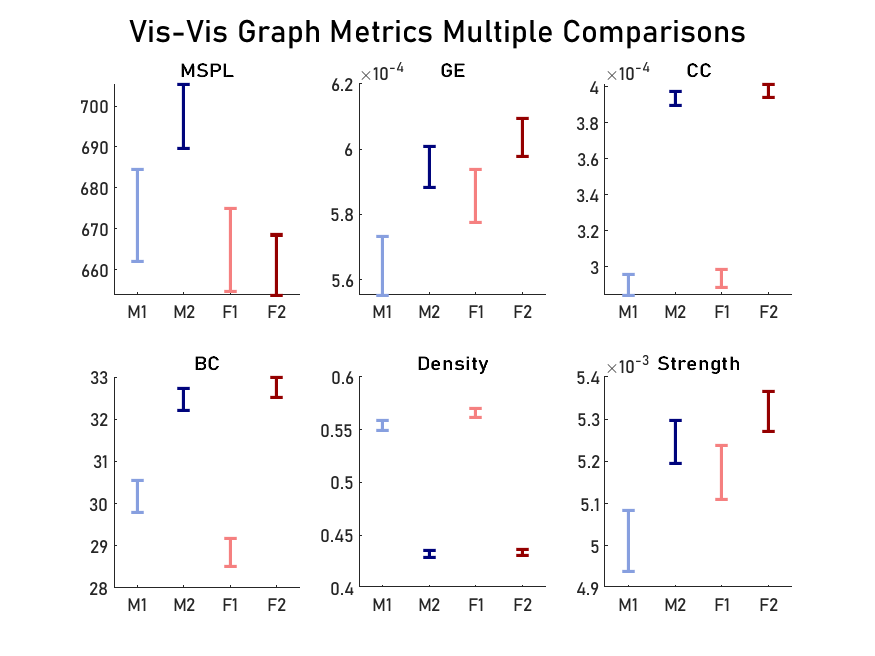


| Vis-Vis Visual Graph Metrics Multiple Comparisons (Mean ± SE) | | | | |
| --- | --- | --- | --- | --- |
| Metric | M1 | M2 | F1 | F2 |
| MSPL | 673 ± 11.3 | 698 ± 7.87 | 665 ± 10.1 | 661 ± 7.37 |
| GE | 0.000564 ± 9e-06 | 0.000594 ± 6.3e-06 | 0.000586 ± 8.08e-06 | 0.000604 ± 5.9e-06 |
| CC | 0.00029 ± 5.59e-06 | 0.000394 ± 3.91e-06 | 0.000293 ± 5.02e-06 | 0.000398 ± 3.66e-06 |
| BC | 30.2 ± 0.376 | 32.5 ± 0.263 | 28.8 ± 0.338 | 32.7 ± 0.247 |
| Density | 0.554 ± 0.00469 | 0.432 ± 0.00328 | 0.566 ± 0.00421 | 0.434 ± 0.00307 |
| Strength | 0.00501 ± 7.16e-05 | 0.00525 ± 5.01e-05 | 0.00517 ± 6.43e-05 | 0.00532 ± 4.69e-05 |


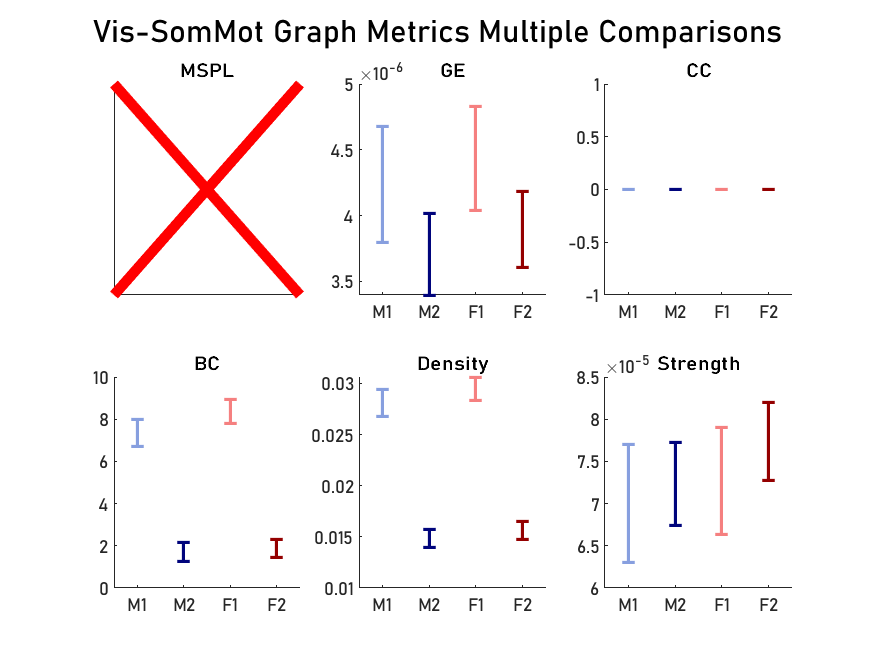


| Vis-SomMot Graph Metrics Multiple Comparisons (Mean ± SE) | | | | |
| --- | --- | --- | --- | --- |
| Metric | M1 | M2 | F1 | F2 |
| MSPL | NaN | NaN | NaN | NaN |
| GE | 4.24e-06 ± 4.41e-07 | 3.7e-06 ± 3.08e-07 | 4.44e-06 ± 3.96e-07 | 3.89e-06 ± 2.89e-07 |
| CC | 0 ± 0 | 0 ± 0 | 0 ± 0 | 0 ± 0 |
| BC | 7.36 ± 0.646 | 1.71 ± 0.452 | 8.37 ± 0.579 | 1.87 ± 0.423 |
| Density | 0.0281 ± 0.00129 | 0.0148 ± 0.000902 | 0.0295 ± 0.00116 | 0.0156 ± 0.000845 |
| Strength | 7e-05 ± 7.01e-06 | 7.23e-05 ± 4.91e-06 | 7.27e-05 ± 6.29e-06 | 7.73e-05 ± 4.59e-06 |


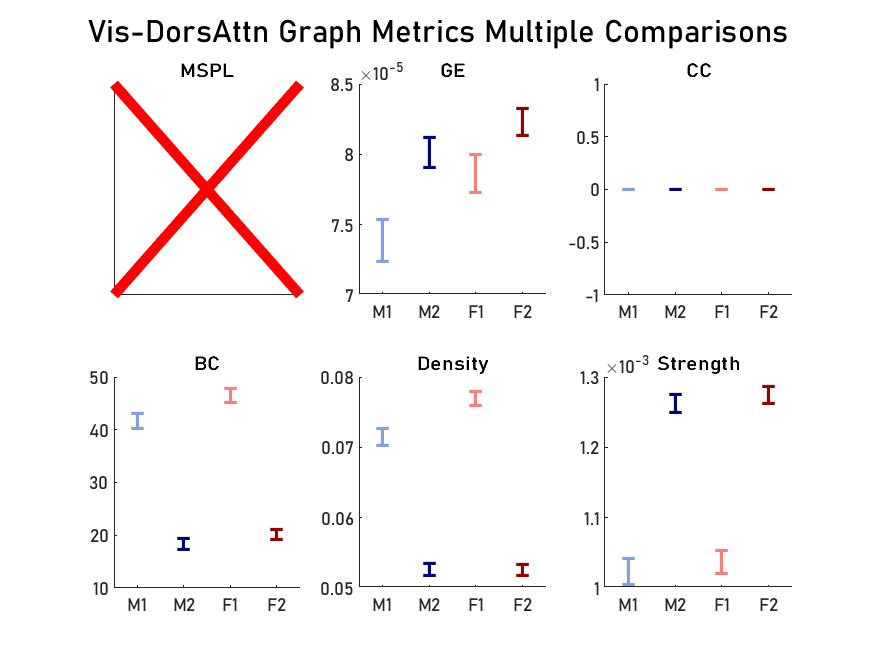


| Vis-DorsAttn Graph Metrics Multiple Comparisons (Mean ± SE) | | | | |
| --- | --- | --- | --- | --- |
| Metric | M1 | M2 | F1 | F2 |
| MSPL | NaN | NaN | NaN | NaN |
| GE | 7.39e-05 ± 1.48e-06 | 8.01e-05 ± 1.04e-06 | 7.87e-05 ± 1.33e-06 | 8.23e-05 ± 9.72e-07 |
| CC | 0 ± 0 | 0 ± 0 | 0 ± 0 | 0 ± 0 |
| BC | 41.6 ± 1.48 | 18.3 ± 1.03 | 46.4 ± 1.33 | 20.2 ± 0.968 |
| Density | 0.0714 ± 0.00118 | 0.0526 ± 0.000828 | 0.0769 ± 0.00106 | 0.0526 ± 0.000776 |
| Strength | 0.00102 ± 1.83e-05 | 0.00126 ± 1.28e-05 | 0.00104 ± 1.65e-05 | 0.00127 ± 1.2e-05 |


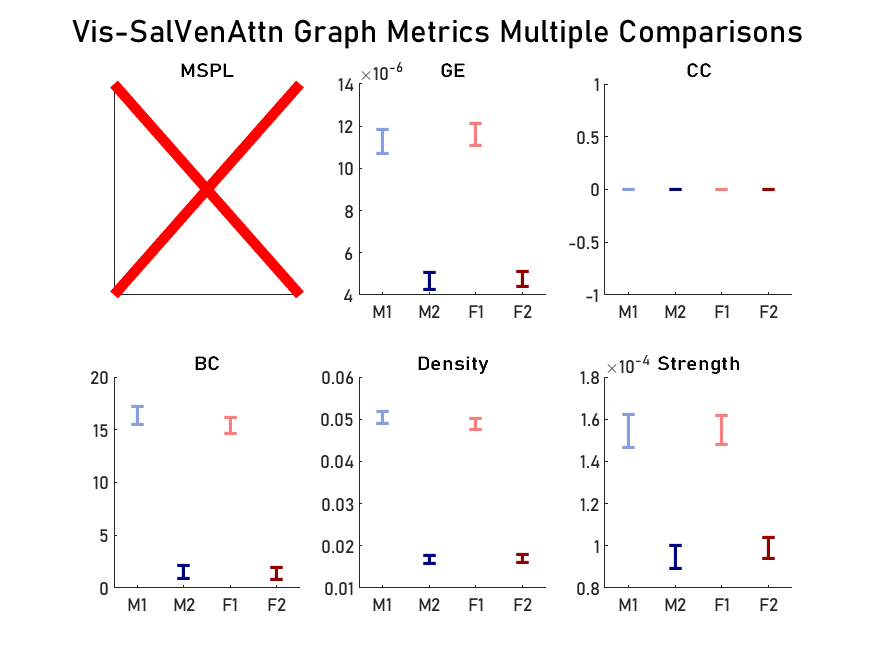


| Vis-SalVenAttn Graph Metrics Multiple Comparisons (Mean ± SE) | | | | |
| --- | --- | --- | --- | --- |
| Metric | M1 | M2 | F1 | F2 |
| MSPL | NaN | NaN | NaN | NaN |
| GE | 1.13e-05 ± 5.66e-07 | 4.66e-06 ± 3.96e-07 | 1.16e-05 ± 5.07e-07 | 4.75e-06 ± 3.71e-07 |
| CC | 0 ± 0 | 0 ± 0 | 0 ± 0 | 0 ± 0 |
| BC | 16.4 ± 0.861 | 1.52 ± 0.602 | 15.4 ± 0.773 | 1.39 ± 0.564 |
| Density | 0.0503 ± 0.00144 | 0.0168 ± 0.00101 | 0.0489 ± 0.00129 | 0.0169 ± 0.000942 |
| Strength | 0.000154 ± 7.7e-06 | 9.48e-05 ± 5.39e-06 | 0.000155 ± 6.91e-06 | 9.9e-05 ± 5.05e-06 |


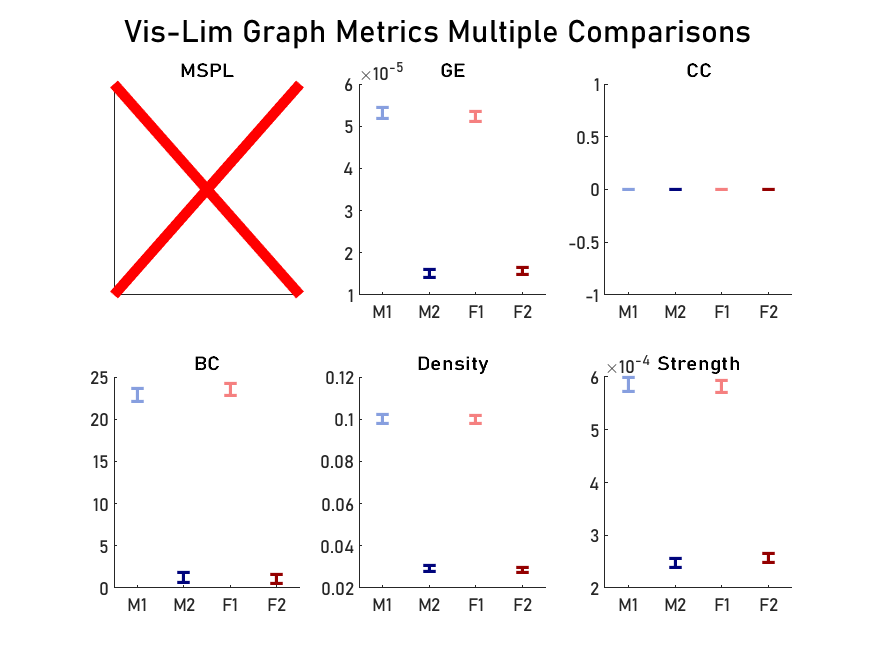


| Vis-Lim Graph Metrics Multiple Comparisons (Mean ± SE) | | | | |
| --- | --- | --- | --- | --- |
| Metric | M1 | M2 | F1 | F2 |
| MSPL | NaN | NaN | NaN | NaN |
| GE | 5.32e-05 ± 1.35e-06 | 1.51e-05 ± 9.46e-07 | 5.23e-05 ± 1.21e-06 | 1.57e-05 ± 8.86e-07 |
| CC | 0 ± 0 | 0 ± 0 | 0 ± 0 | 0 ± 0 |
| BC | 22.9 ± 0.794 | 1.26 ± 0.555 | 23.6 ± 0.712 | 1.08 ± 0.52 |
| Density | 0.1 ± 0.00203 | 0.0291 ± 0.00142 | 0.1 ± 0.00182 | 0.0285 ± 0.00133 |
| Strength | 0.000586 ± 1.31e-05 | 0.000247 ± 9.18e-06 | 0.000582 ± 1.18e-05 | 0.000257 ± 8.6e-06 |


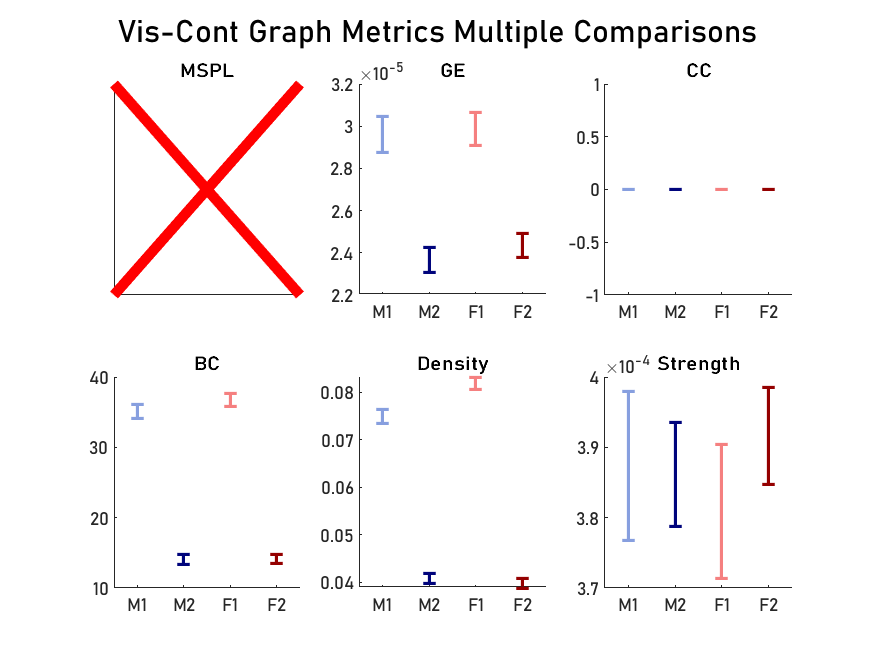


| Vis-Cont Graph Metrics Multiple Comparisons (Mean ± SE) | | | | |
| --- | --- | --- | --- | --- |
| Metric | M1 | M2 | F1 | F2 |
| MSPL | NaN | NaN | NaN | NaN |
| GE | 2.96e-05 ± 8.67e-07 | 2.37e-05 ± 6.07e-07 | 2.99e-05 ± 7.78e-07 | 2.43e-05 ± 5.68e-07 |
| CC | 0 ± 0 | 0 ± 0 | 0 ± 0 | 0 ± 0 |
| BC | 35.1 ± 1.02 | 14 ± 0.715 | 36.7 ± 0.917 | 14.1 ± 0.67 |
| Density | 0.075 ± 0.00149 | 0.0408 ± 0.00104 | 0.0819 ± 0.00134 | 0.0398 ± 0.000977 |
| Strength | 0.000387 ± 1.05e-05 | 0.000386 ± 7.38e-06 | 0.000381 ± 9.46e-06 | 0.000392 ± 6.91e-06 |


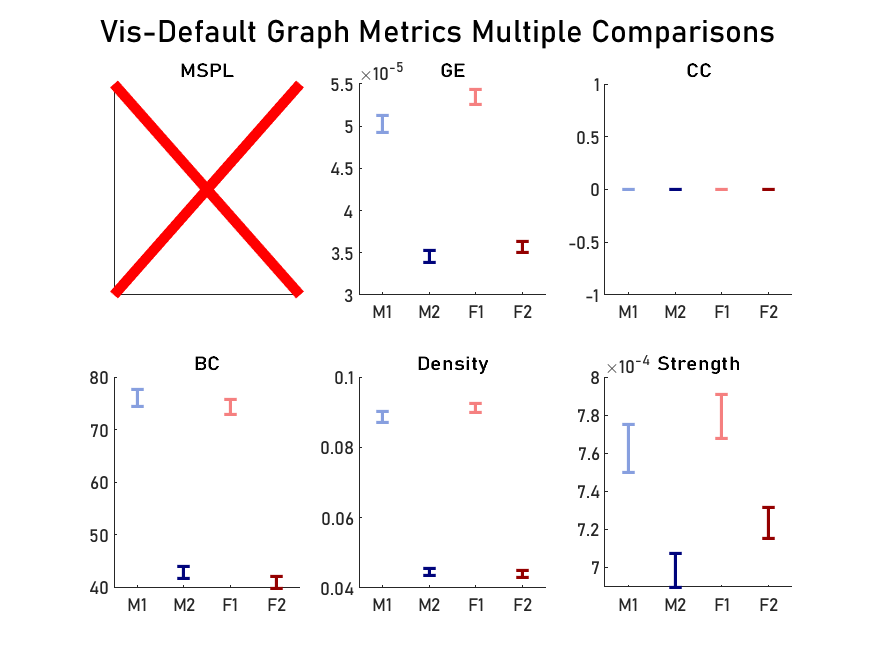


| Vis-Default Graph Metrics Multiple Comparisons (Mean ± SE) | | | | |
| --- | --- | --- | --- | --- |
| Metric | M1 | M2 | F1 | F2 |
| MSPL | NaN | NaN | NaN | NaN |
| GE | 5.02e-05 ± 1e-06 | 3.45e-05 ± 7e-07 | 5.34e-05 ± 8.98e-07 | 3.56e-05 ± 6.56e-07 |
| CC | 0 ± 0 | 0 ± 0 | 0 ± 0 | 0 ± 0 |
| BC | 75.9 ± 1.62 | 42.9 ± 1.13 | 74.3 ± 1.45 | 40.9 ± 1.06 |
| Density | 0.0886 ± 0.00149 | 0.0446 ± 0.00104 | 0.0911 ± 0.00133 | 0.0441 ± 0.000974 |
| Strength | 0.000762 ± 1.28e-05 | 0.000698 ± 8.94e-06 | 0.000779 ± 1.15e-05 | 0.000723 ± 8.38e-06 |


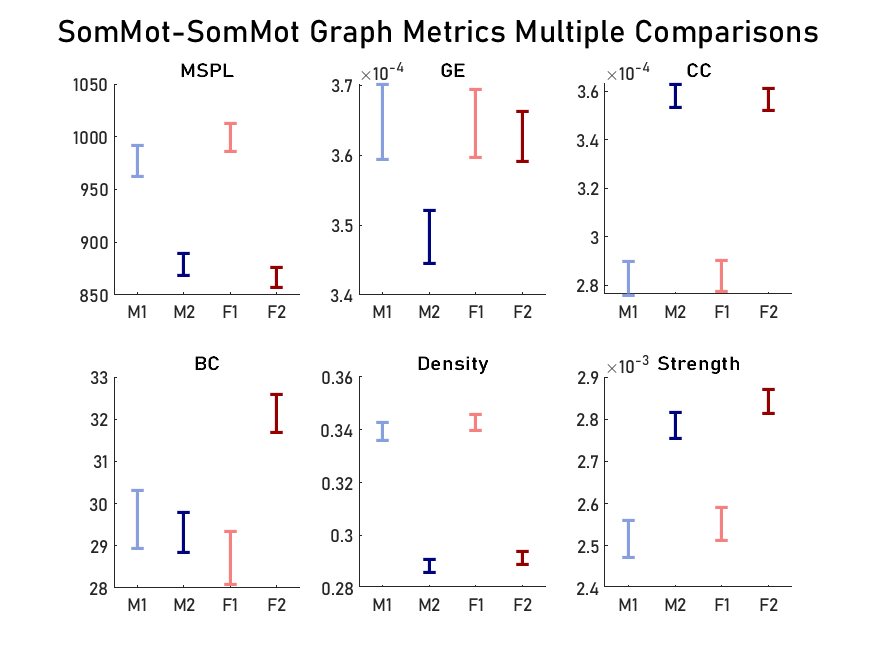


| SomMot-SomMot Graph Metrics Multiple Comparisons (Mean ± SE) | | | | |
| --- | --- | --- | --- | --- |
| Metric | M1 | M2 | F1 | F2 |
| MSPL | 977 ± 14.8 | 879 ± 10.4 | 999 ± 13.3 | 867 ± 9.71 |
| GE | 0.000365 ± 5.42e-06 | 0.000348 ± 3.79e-06 | 0.000365 ± 4.87e-06 | 0.000363 ± 3.55e-06 |
| CC | 0.000283 ± 6.97e-06 | 0.000358 ± 4.88e-06 | 0.000284 ± 6.26e-06 | 0.000357 ± 4.57e-06 |
| BC | 29.6 ± 0.689 | 29.3 ± 0.482 | 28.7 ± 0.619 | 32.1 ± 0.452 |
| Density | 0.339 ± 0.00352 | 0.288 ± 0.00247 | 0.343 ± 0.00316 | 0.291 ± 0.00231 |
| Strength | 0.00252 ± 4.38e-05 | 0.00278 ± 3.06e-05 | 0.00255 ± 3.93e-05 | 0.00284 ± 2.87e-05 |


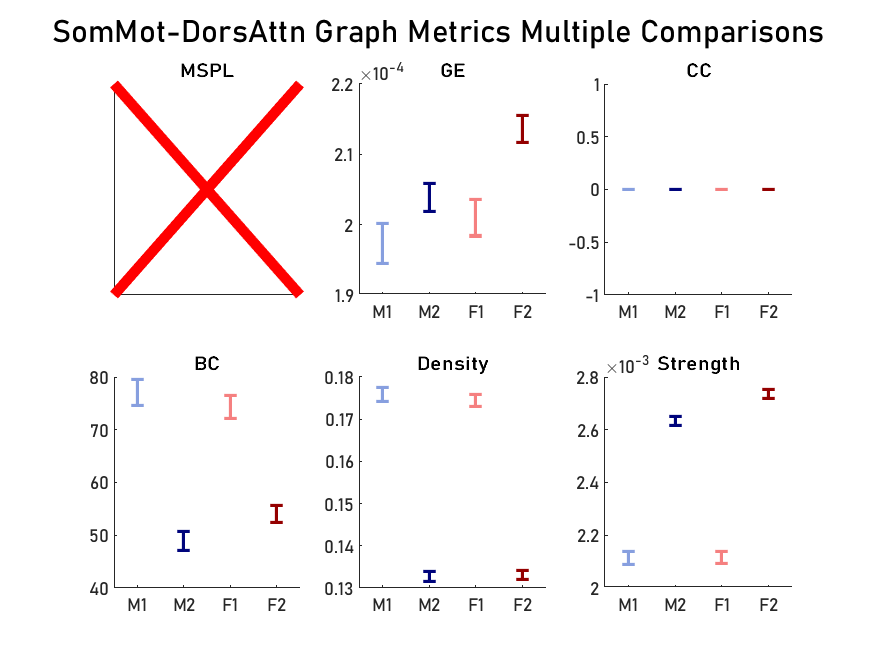


| SomMot-DorsAttn Graph Metrics Multiple Comparisons (Mean ± SE) | | | | |
| --- | --- | --- | --- | --- |
| Metric | M1 | M2 | F1 | F2 |
| MSPL | NaN | NaN | NaN | NaN |
| GE | 0.000197 ± 2.89e-06 | 0.000204 ± 2.02e-06 | 0.000201 ± 2.59e-06 | 0.000214 ± 1.89e-06 |
| CC | 0 ± 0 | 0 ± 0 | 0 ± 0 | 0 ± 0 |
| BC | 77.1 ± 2.52 | 48.9 ± 1.77 | 74.3 ± 2.26 | 54 ± 1.65 |
| Density | 0.176 ± 0.00169 | 0.133 ± 0.00119 | 0.174 ± 0.00152 | 0.133 ± 0.00111 |
| Strength | 0.00211 ± 2.6e-05 | 0.00263 ± 1.82e-05 | 0.00212 ± 2.33e-05 | 0.00274 ± 1.7e-05 |


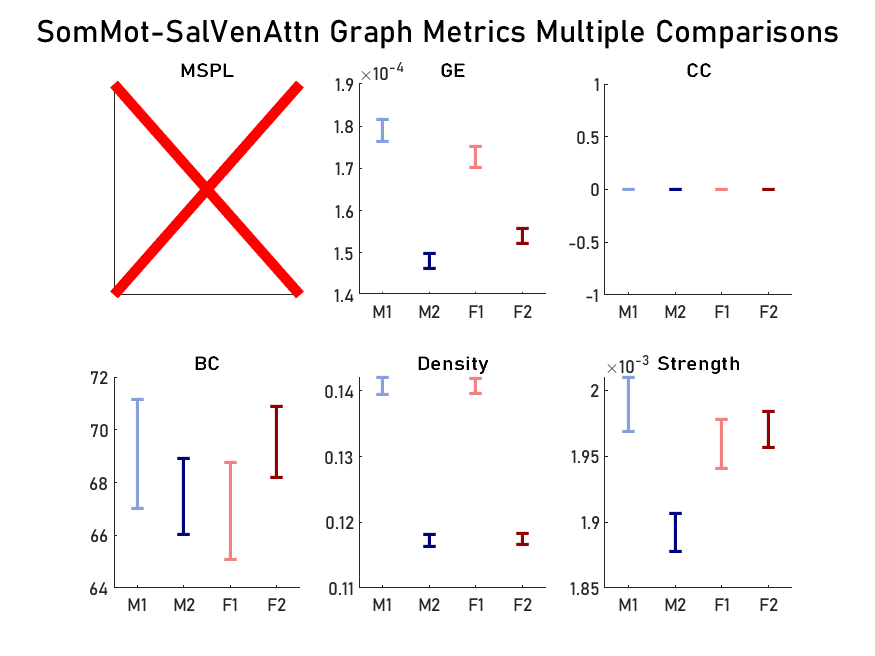


| SomMot-SalVenAttn Graph Metrics Multiple Comparisons (Mean ± SE) | | | | |
| --- | --- | --- | --- | --- |
| Metric | M1 | M2 | F1 | F2 |
| MSPL | NaN | NaN | NaN | NaN |
| GE | 0.000179 ± 2.67e-06 | 0.000148 ± 1.87e-06 | 0.000173 ± 2.4e-06 | 0.000154 ± 1.75e-06 |
| CC | 0 ± 0 | 0 ± 0 | 0 ± 0 | 0 ± 0 |
| BC | 69.1 ± 2.06 | 67.5 ± 1.44 | 66.9 ± 1.84 | 69.5 ± 1.35 |
| Density | 0.141 ± 0.00131 | 0.117 ± 0.000914 | 0.141 ± 0.00117 | 0.117 ± 0.000856 |
| Strength | 0.00199 ± 2.08e-05 | 0.00189 ± 1.45e-05 | 0.00196 ± 1.87e-05 | 0.00197 ± 1.36e-05 |


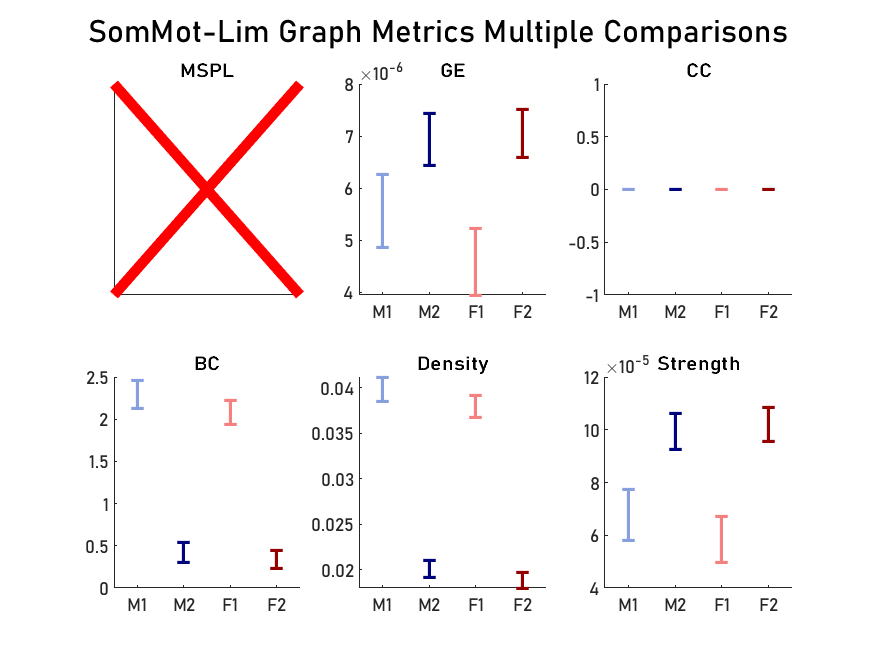


| SomMot-Lim Graph Metrics Multiple Comparisons (Mean ± SE) | | | | |
| --- | --- | --- | --- | --- |
| Metric | M1 | M2 | F1 | F2 |
| MSPL | NaN | NaN | NaN | NaN |
| GE | 5.56e-06 ± 7.09e-07 | 6.93e-06 ± 4.96e-07 | 4.58e-06 ± 6.36e-07 | 7.05e-06 ± 4.65e-07 |
| CC | 0 ± 0 | 0 ± 0 | 0 ± 0 | 0 ± 0 |
| BC | 2.29 ± 0.162 | 0.42 ± 0.113 | 2.08 ± 0.146 | 0.333 ± 0.106 |
| Density | 0.0398 ± 0.00134 | 0.0201 ± 0.000937 | 0.0379 ± 0.0012 | 0.0189 ± 0.000878 |
| Strength | 6.77e-05 ± 9.7e-06 | 9.93e-05 ± 6.78e-06 | 5.86e-05 ± 8.7e-06 | 0.000102 ± 6.35e-06 |


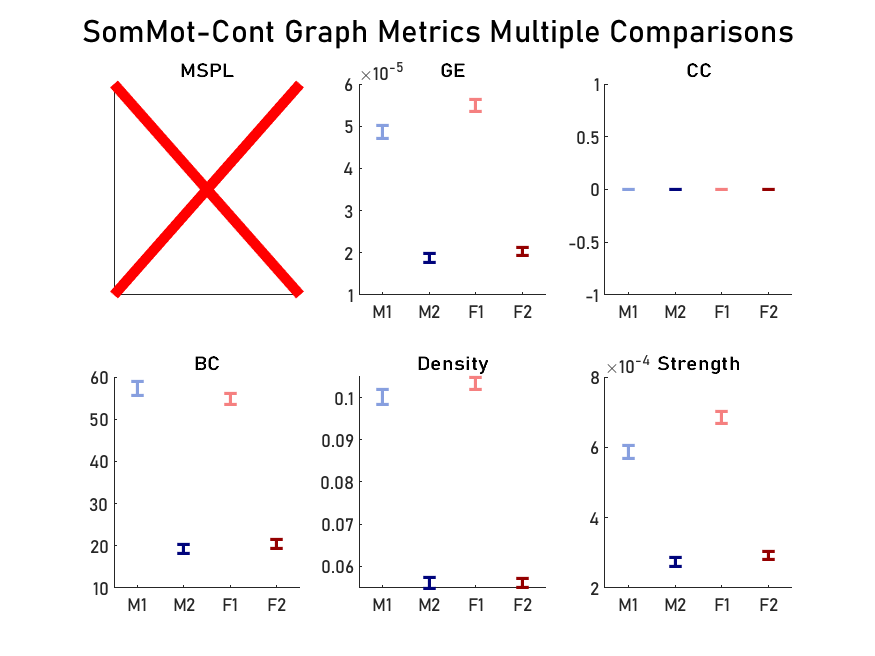


| SomMot-Cont Graph Metrics Multiple Comparisons (Mean ± SE) | | | | |
| --- | --- | --- | --- | --- |
| Metric | M1 | M2 | F1 | F2 |
| MSPL | NaN | NaN | NaN | NaN |
| GE | 4.87e-05 ± 1.53e-06 | 1.87e-05 ± 1.07e-06 | 5.49e-05 ± 1.37e-06 | 2.03e-05 ± 1e-06 |
| CC | 0 ± 0 | 0 ± 0 | 0 ± 0 | 0 ± 0 |
| BC | 57.3 ± 1.56 | 19.2 ± 1.09 | 54.8 ± 1.4 | 20.4 ± 1.02 |
| Density | 0.1 ± 0.00172 | 0.056 ± 0.0012 | 0.103 ± 0.00154 | 0.056 ± 0.00113 |
| Strength | 0.000587 ± 1.82e-05 | 0.000273 ± 1.27e-05 | 0.000685 ± 1.63e-05 | 0.000293 ± 1.19e-05 |


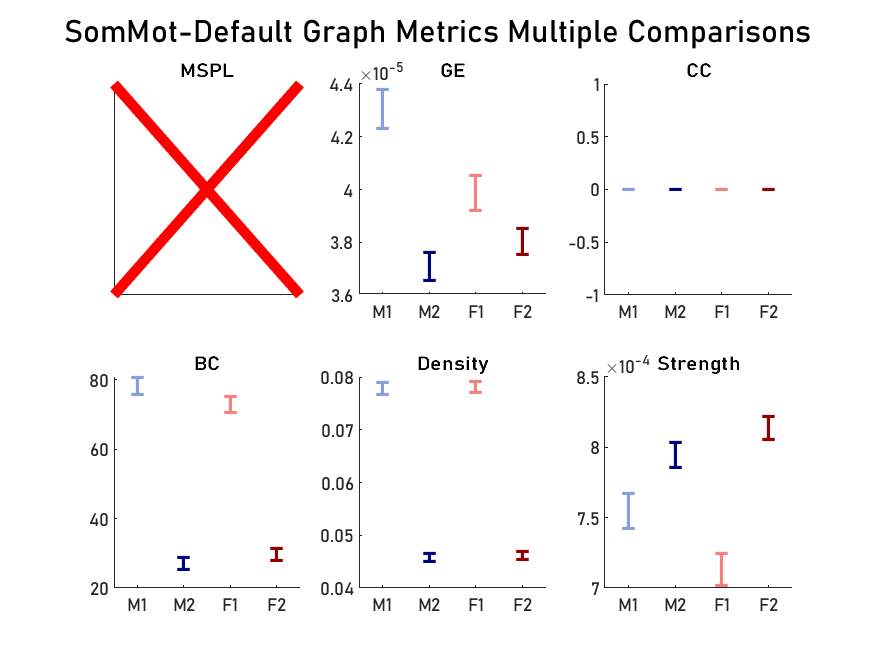


| SomMot-Default Graph Metrics Multiple Comparisons (Mean ± SE) | | | | |
| --- | --- | --- | --- | --- |
| Metric | M1 | M2 | F1 | F2 |
| MSPL | NaN | NaN | NaN | NaN |
| GE | 4.3e-05 ± 7.46e-07 | 3.71e-05 ± 5.22e-07 | 3.99e-05 ± 6.69e-07 | 3.8e-05 ± 4.89e-07 |
| CC | 0 ± 0 | 0 ± 0 | 0 ± 0 | 0 ± 0 |
| BC | 78.3 ± 2.53 | 27.1 ± 1.77 | 72.9 ± 2.27 | 29.7 ± 1.66 |
| Density | 0.0779 ± 0.00114 | 0.0458 ± 0.000795 | 0.0781 ± 0.00102 | 0.0462 ± 0.000744 |
| Strength | 0.000755 ± 1.25e-05 | 0.000794 ± 8.74e-06 | 0.000713 ± 1.12e-05 | 0.000814 ± 8.19e-06 |


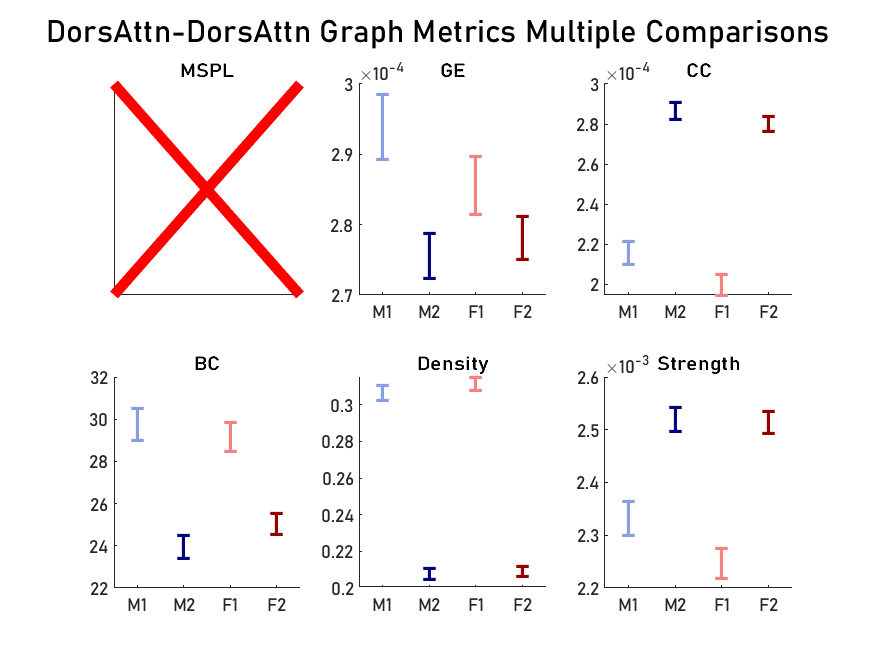


| DorsAttn-DorsAttn Graph Metrics Multiple Comparisons (Mean ± SE) | | | | |
| --- | --- | --- | --- | --- |
| Metric | M1 | M2 | F1 | F2 |
| MSPL | NaN | NaN | NaN | NaN |
| GE | 0.000294 ± 4.62e-06 | 0.000276 ± 3.23e-06 | 0.000286 ± 4.15e-06 | 0.000278 ± 3.03e-06 |
| CC | 0.000216 ± 5.76e-06 | 0.000287 ± 4.03e-06 | 0.0002 ± 5.16e-06 | 0.00028 ± 3.77e-06 |
| BC | 29.7 ± 0.765 | 23.9 ± 0.535 | 29.1 ± 0.686 | 25 ± 0.501 |
| Density | 0.307 ± 0.00408 | 0.208 ± 0.00286 | 0.312 ± 0.00366 | 0.209 ± 0.00267 |
| Strength | 0.00233 ± 3.22e-05 | 0.00252 ± 2.25e-05 | 0.00225 ± 2.89e-05 | 0.00251 ± 2.11e-05 |


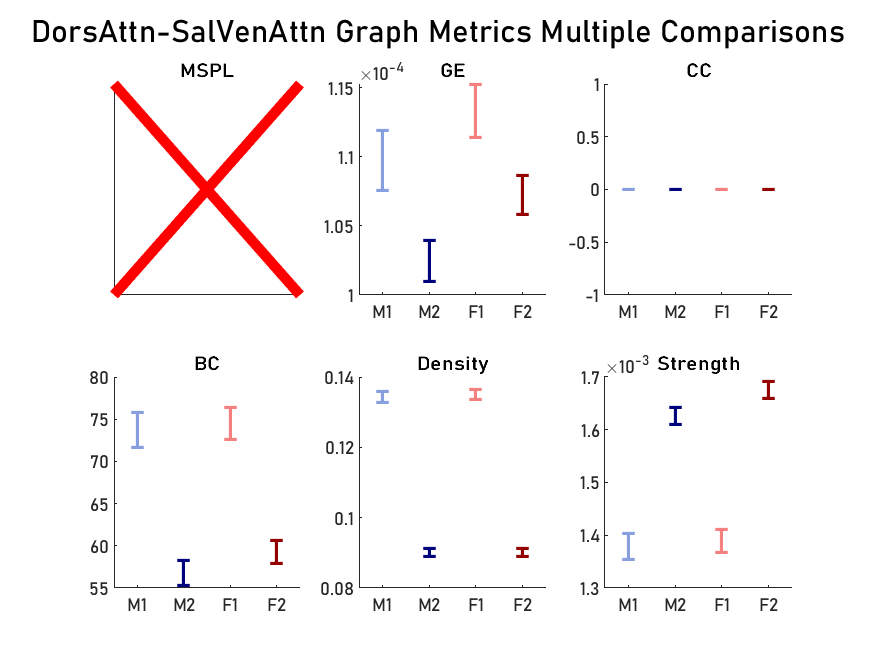


| DorsAttn-SalVenAttn Graph Metrics Multiple Comparisons (Mean ± SE) | | | | |
| --- | --- | --- | --- | --- |
| Metric | M1 | M2 | F1 | F2 |
| MSPL | NaN | NaN | NaN | NaN |
| GE | 0.00011 ± 2.15e-06 | 0.000102 ± 1.51e-06 | 0.000113 ± 1.93e-06 | 0.000107 ± 1.41e-06 |
| CC | 0 ± 0 | 0 ± 0 | 0 ± 0 | 0 ± 0 |
| BC | 73.7 ± 2.11 | 56.7 ± 1.48 | 74.5 ± 1.89 | 59.2 ± 1.38 |
| Density | 0.134 ± 0.00167 | 0.0902 ± 0.00116 | 0.135 ± 0.00149 | 0.0902 ± 0.00109 |
| Strength | 0.00138 ± 2.42e-05 | 0.00163 ± 1.69e-05 | 0.00139 ± 2.17e-05 | 0.00168 ± 1.59e-05 |


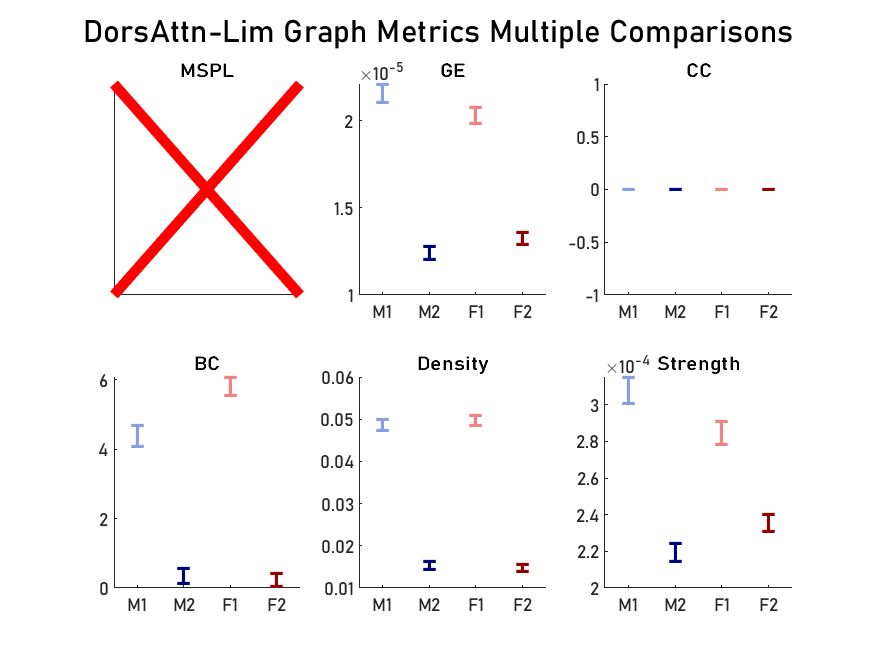


| DorsAttn-Lim Graph Metrics Multiple Comparisons (Mean ± SE) | | | | |
| --- | --- | --- | --- | --- |
| Metric | M1 | M2 | F1 | F2 |
| MSPL | NaN | NaN | NaN | NaN |
| GE | 2.16e-05 ± 5.18e-07 | 1.24e-05 ± 3.62e-07 | 2.03e-05 ± 4.65e-07 | 1.32e-05 ± 3.39e-07 |
| CC | 0 ± 0 | 0 ± 0 | 0 ± 0 | 0 ± 0 |
| BC | 4.39 ± 0.304 | 0.346 ± 0.213 | 5.8 ± 0.273 | 0.229 ± 0.199 |
| Density | 0.0486 ± 0.0013 | 0.0153 ± 0.000913 | 0.0498 ± 0.00117 | 0.0147 ± 0.000855 |
| Strength | 0.000308 ± 7.15e-06 | 0.00022 ± 5e-06 | 0.000285 ± 6.42e-06 | 0.000236 ± 4.69e-06 |


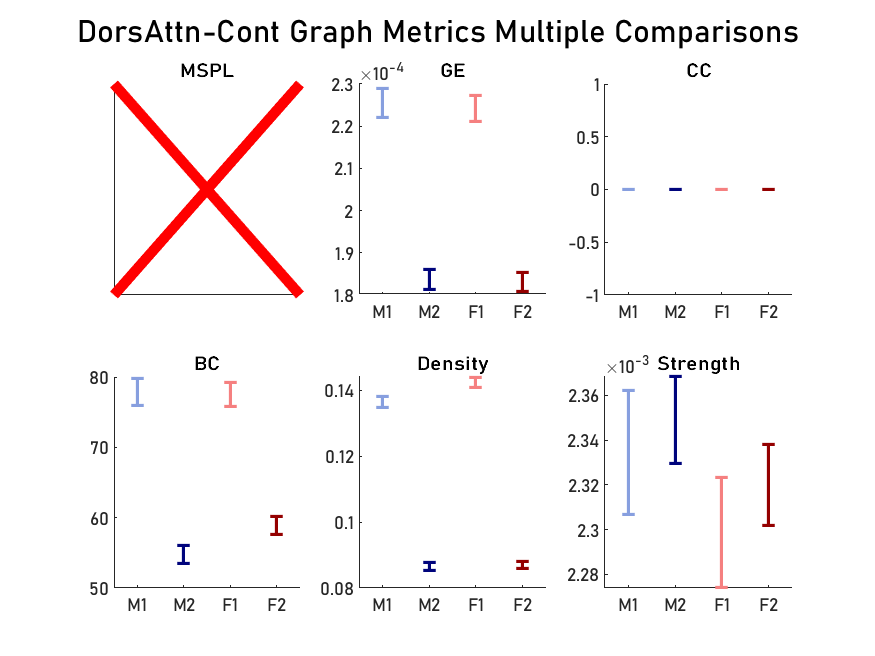


| DorsAttn-Cont Graph Metrics Multiple Comparisons (Mean ± SE) | | | | |
| --- | --- | --- | --- | --- |
| Metric | M1 | M2 | F1 | F2 |
| MSPL | NaN | NaN | NaN | NaN |
| GE | 0.000226 ± 3.45e-06 | 0.000184 ± 2.41e-06 | 0.000224 ± 3.09e-06 | 0.000183 ± 2.26e-06 |
| CC | 0 ± 0 | 0 ± 0 | 0 ± 0 | 0 ± 0 |
| BC | 77.9 ± 1.91 | 54.8 ± 1.34 | 77.5 ± 1.71 | 58.9 ± 1.25 |
| Density | 0.136 ± 0.00177 | 0.0864 ± 0.00124 | 0.142 ± 0.00158 | 0.087 ± 0.00116 |
| Strength | 0.00233 ± 2.76e-05 | 0.00235 ± 1.93e-05 | 0.0023 ± 2.48e-05 | 0.00232 ± 1.81e-05 |


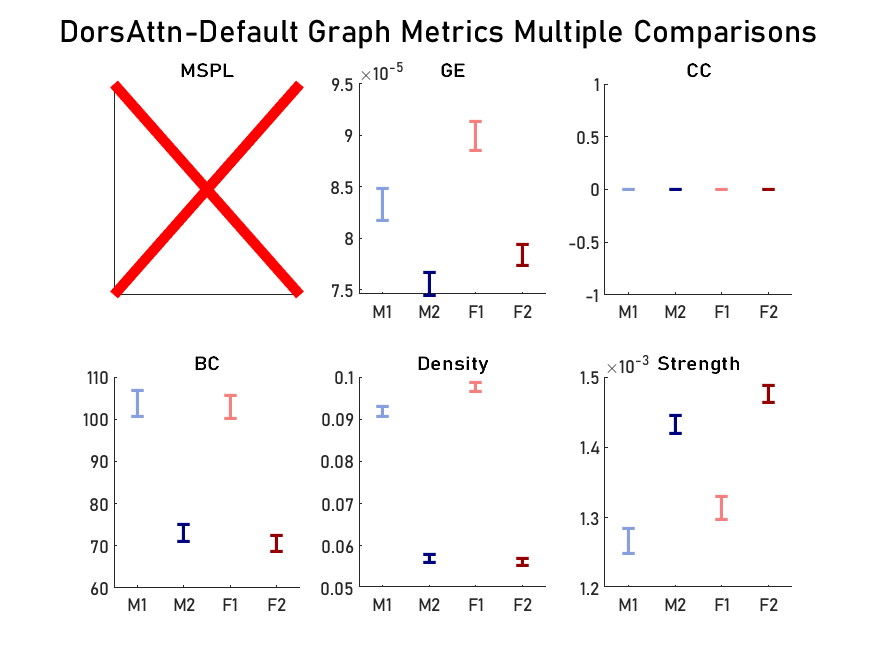


| DorsAttn-Default Graph Metrics Multiple Comparisons (Mean ± SE) | | | | |
| --- | --- | --- | --- | --- |
| Metric | M1 | M2 | F1 | F2 |
| MSPL | NaN | NaN | NaN | NaN |
| GE | 8.33e-05 ± 1.57e-06 | 7.56e-05 ± 1.1e-06 | 8.99e-05 ± 1.4e-06 | 7.84e-05 ± 1.03e-06 |
| CC | 0 ± 0 | 0 ± 0 | 0 ± 0 | 0 ± 0 |
| BC | 104 ± 2.99 | 73 ± 2.09 | 103 ± 2.68 | 70.6 ± 1.96 |
| Density | 0.0918 ± 0.00126 | 0.057 ± 0.00088 | 0.0977 ± 0.00113 | 0.0562 ± 0.000824 |
| Strength | 0.00127 ± 1.79e-05 | 0.00143 ± 1.26e-05 | 0.00131 ± 1.61e-05 | 0.00148 ± 1.18e-05 |


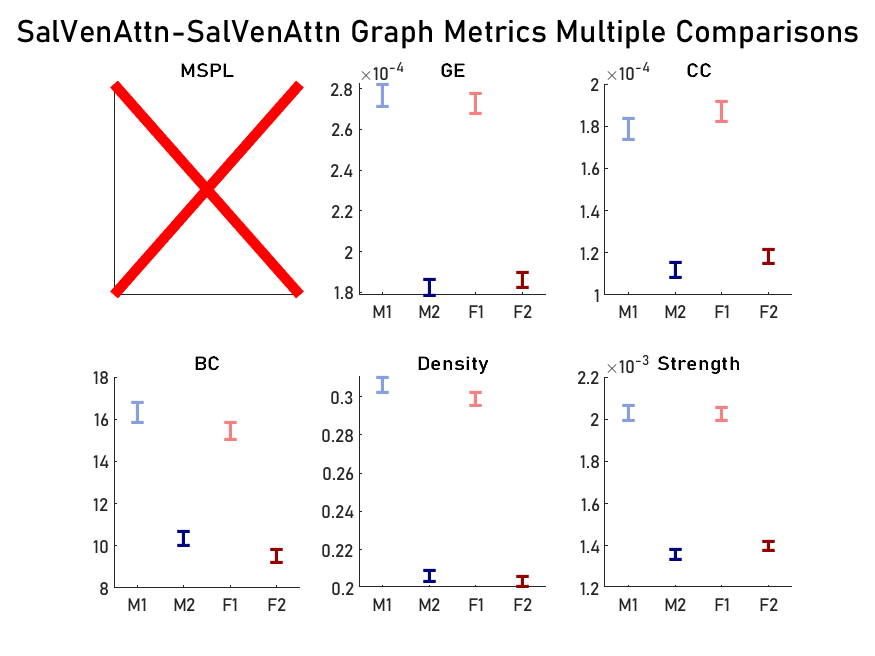


| SalVenAttn-SalVenAttn Graph Metrics Multiple Comparisons (Mean ± SE) | | | | |
| --- | --- | --- | --- | --- |
| Metric | M1 | M2 | F1 | F2 |
| MSPL | NaN | NaN | NaN | NaN |
| GE | 0.000277 ± 5.6e-06 | 0.000183 ± 3.92e-06 | 0.000273 ± 5.02e-06 | 0.000186 ± 3.67e-06 |
| CC | 0.000179 ± 5.11e-06 | 0.000112 ± 3.57e-06 | 0.000187 ± 4.58e-06 | 0.000118 ± 3.35e-06 |
| BC | 16.3 ± 0.466 | 10.4 ± 0.326 | 15.4 ± 0.418 | 9.54 ± 0.305 |
| Density | 0.306 ± 0.00398 | 0.206 ± 0.00278 | 0.299 ± 0.00357 | 0.204 ± 0.00261 |
| Strength | 0.00203 ± 3.33e-05 | 0.00136 ± 2.33e-05 | 0.00202 ± 2.99e-05 | 0.0014 ± 2.18e-05 |


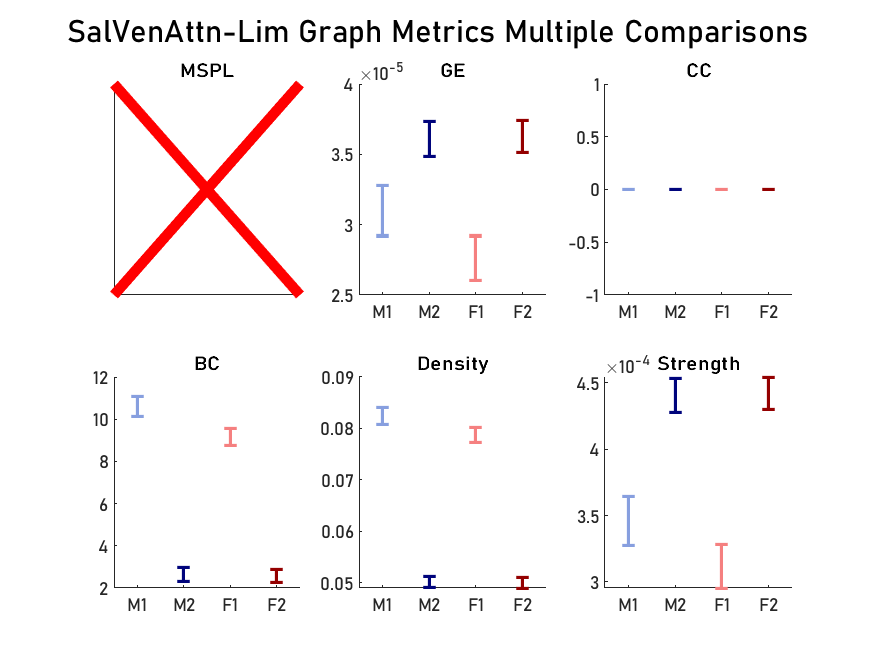


| SalVenAttn-Lim Graph Metrics Multiple Comparisons (Mean ± SE) | | | | |
| --- | --- | --- | --- | --- |
| Metric | M1 | M2 | F1 | F2 |
| MSPL | NaN | NaN | NaN | NaN |
| GE | 3.1e-05 ± 1.79e-06 | 3.61e-05 ± 1.25e-06 | 2.76e-05 ± 1.6e-06 | 3.63e-05 ± 1.17e-06 |
| CC | 0 ± 0 | 0 ± 0 | 0 ± 0 | 0 ± 0 |
| BC | 10.6 ± 0.462 | 2.65 ± 0.323 | 9.17 ± 0.414 | 2.57 ± 0.303 |
| Density | 0.0824 ± 0.00162 | 0.0501 ± 0.00114 | 0.0788 ± 0.00146 | 0.05 ± 0.00106 |
| Strength | 0.000346 ± 1.84e-05 | 0.000441 ± 1.29e-05 | 0.000312 ± 1.65e-05 | 0.000442 ± 1.21e-05 |


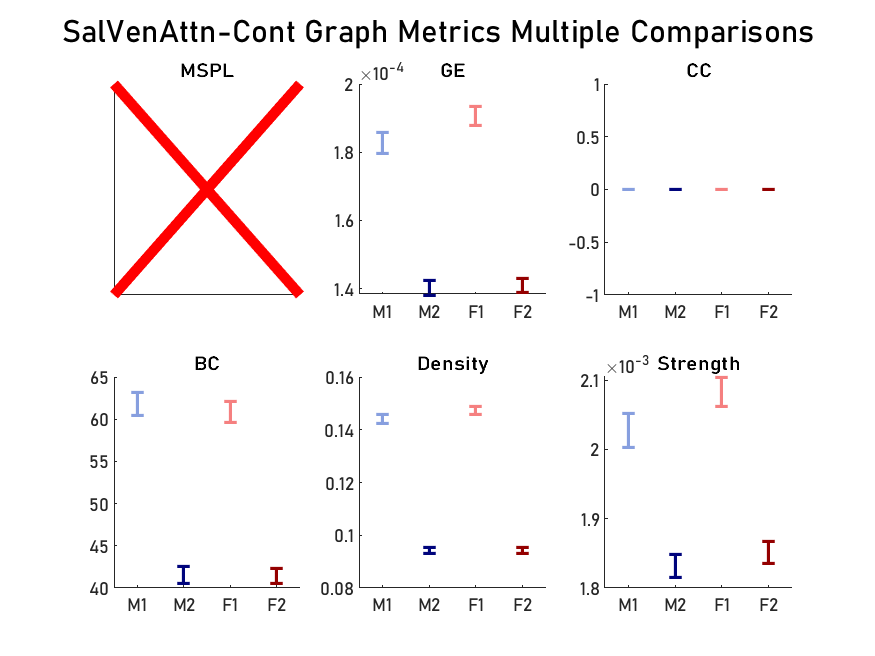


| SalVenAttn-Cont Graph Metrics Multiple Comparisons (Mean ± SE) | | | | |
| --- | --- | --- | --- | --- |
| Metric | M1 | M2 | F1 | F2 |
| MSPL | NaN | NaN | NaN | NaN |
| GE | 0.000183 ± 3.1e-06 | 0.00014 ± 2.17e-06 | 0.000191 ± 2.78e-06 | 0.000141 ± 2.03e-06 |
| CC | 0 ± 0 | 0 ± 0 | 0 ± 0 | 0 ± 0 |
| BC | 61.8 ± 1.38 | 41.6 ± 0.967 | 60.9 ± 1.24 | 41.4 ± 0.906 |
| Density | 0.144 ± 0.00181 | 0.0942 ± 0.00127 | 0.147 ± 0.00162 | 0.0942 ± 0.00119 |
| Strength | 0.00203 ± 2.4e-05 | 0.00183 ± 1.68e-05 | 0.00208 ± 2.15e-05 | 0.00185 ± 1.57e-05 |


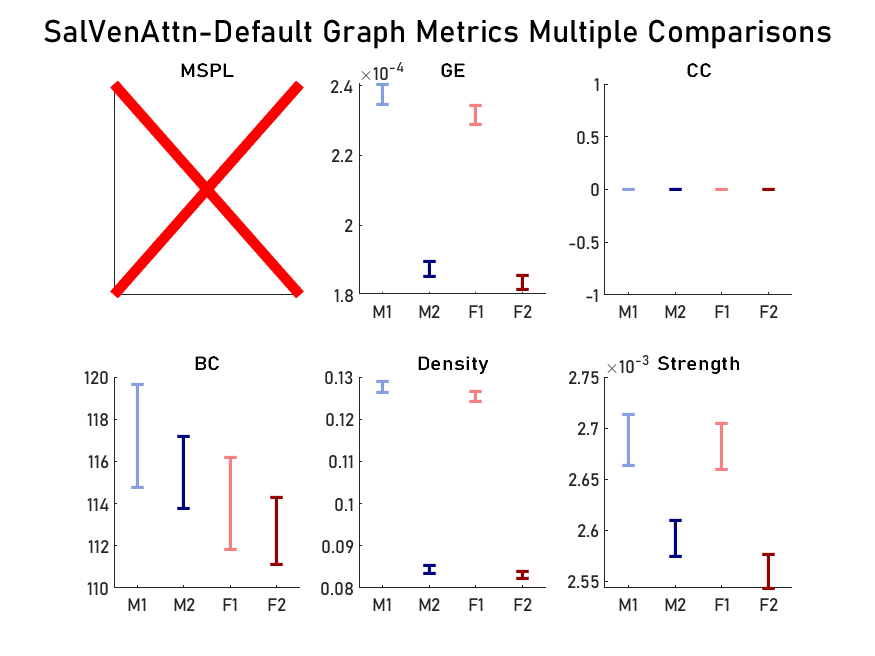


| SalVenAttn-Default Graph Metrics Multiple Comparisons (Mean ± SE) | | | | |
| --- | --- | --- | --- | --- |
| Metric | M1 | M2 | F1 | F2 |
| MSPL | NaN | NaN | NaN | NaN |
| GE | 0.000238 ± 2.96e-06 | 0.000187 ± 2.07e-06 | 0.000232 ± 2.66e-06 | 0.000184 ± 1.94e-06 |
| CC | 0 ± 0 | 0 ± 0 | 0 ± 0 | 0 ± 0 |
| BC | 117 ± 2.43 | 115 ± 1.7 | 114 ± 2.18 | 113 ± 1.59 |
| Density | 0.128 ± 0.00127 | 0.0844 ± 0.000887 | 0.125 ± 0.00114 | 0.083 ± 0.000831 |
| Strength | 0.00269 ± 2.52e-05 | 0.00259 ± 1.76e-05 | 0.00268 ± 2.26e-05 | 0.00256 ± 1.65e-05 |


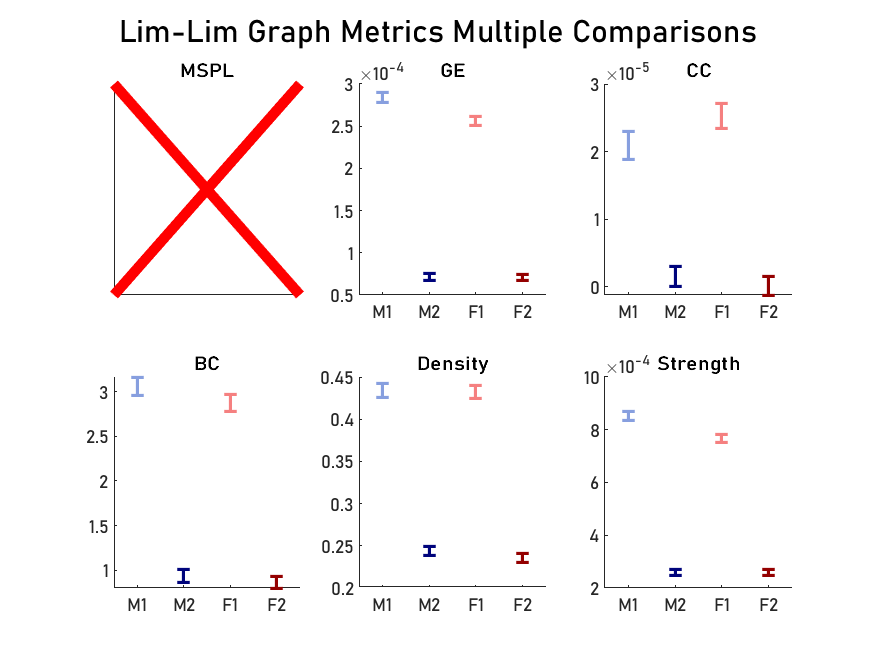


| Lim-Lim Graph Metrics Multiple Comparisons (Mean ± SE) | | | | |
| --- | --- | --- | --- | --- |
| Metric | M1 | M2 | F1 | F2 |
| MSPL | NaN | NaN | NaN | NaN |
| GE | 0.000284 ± 5.91e-06 | 7.12e-05 ± 4.13e-06 | 0.000256 ± 5.3e-06 | 7.05e-05 ± 3.87e-06 |
| CC | 2.09e-05 ± 2.06e-06 | 1.56e-06 ± 1.44e-06 | 2.52e-05 ± 1.85e-06 | 1.52e-07 ± 1.35e-06 |
| BC | 3.06 ± 0.102 | 0.937 ± 0.0716 | 2.87 ± 0.0918 | 0.867 ± 0.0671 |
| Density | 0.434 ± 0.00826 | 0.244 ± 0.00578 | 0.432 ± 0.00741 | 0.235 ± 0.00541 |
| Strength | 0.000852 ± 1.7e-05 | 0.000259 ± 1.19e-05 | 0.000765 ± 1.52e-05 | 0.000259 ± 1.11e-05 |


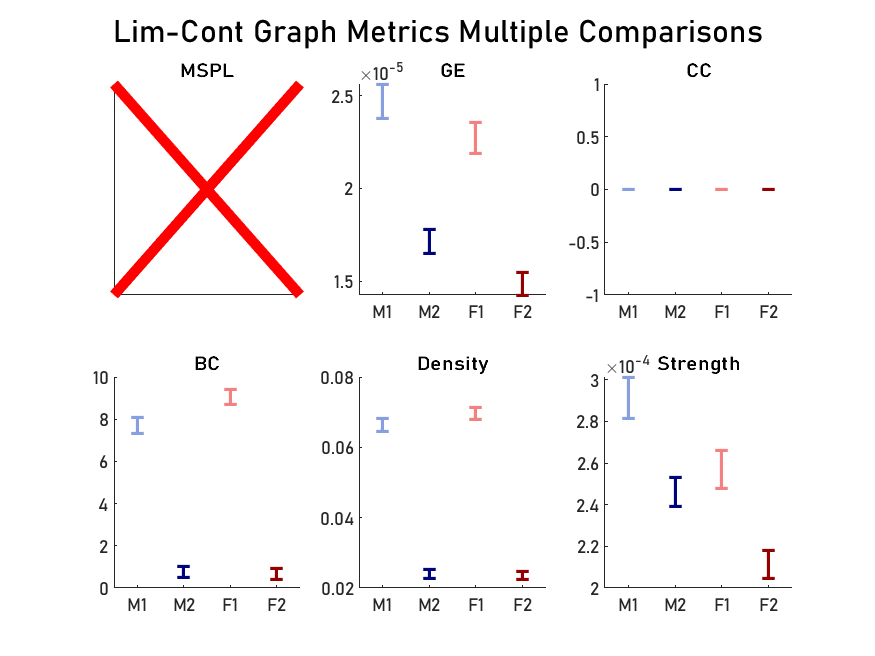


| Lim-Cont Graph Metrics Multiple Comparisons (Mean ± SE) | | | | |
| --- | --- | --- | --- | --- |
| Metric | M1 | M2 | F1 | F2 |
| MSPL | NaN | NaN | NaN | NaN |
| GE | 2.47e-05 ± 9.24e-07 | 1.72e-05 ± 6.47e-07 | 2.27e-05 ± 8.29e-07 | 1.49e-05 ± 6.06e-07 |
| CC | 0 ± 0 | 0 ± 0 | 0 ± 0 | 0 ± 0 |
| BC | 7.69 ± 0.38 | 0.74 ± 0.266 | 9.05 ± 0.341 | 0.67 ± 0.249 |
| Density | 0.0664 ± 0.0018 | 0.0241 ± 0.00126 | 0.0696 ± 0.00161 | 0.0236 ± 0.00118 |
| Strength | 0.000291 ± 9.99e-06 | 0.000246 ± 6.99e-06 | 0.000257 ± 8.96e-06 | 0.000211 ± 6.54e-06 |


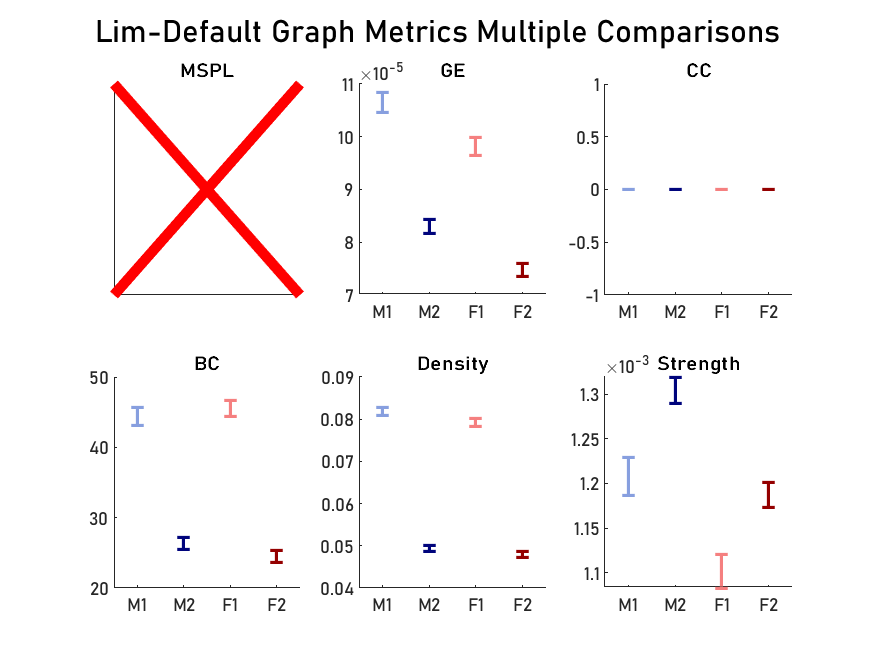


| Lim-Default Graph Metrics Multiple Comparisons (Mean ± SE) | | | | |
| --- | --- | --- | --- | --- |
| Metric | M1 | M2 | F1 | F2 |
| MSPL | NaN | NaN | NaN | NaN |
| GE | 0.000107 ± 1.9e-06 | 8.31e-05 ± 1.33e-06 | 9.82e-05 ± 1.7e-06 | 7.48e-05 ± 1.24e-06 |
| CC | 0 ± 0 | 0 ± 0 | 0 ± 0 | 0 ± 0 |
| BC | 44.4 ± 1.29 | 26.3 ± 0.9 | 45.5 ± 1.15 | 24.5 ± 0.843 |
| Density | 0.0818 ± 0.00103 | 0.0494 ± 0.000717 | 0.0792 ± 0.00092 | 0.048 ± 0.000672 |
| Strength | 0.00121 ± 2.1e-05 | 0.0013 ± 1.47e-05 | 0.0011 ± 1.89e-05 | 0.00119 ± 1.38e-05 |


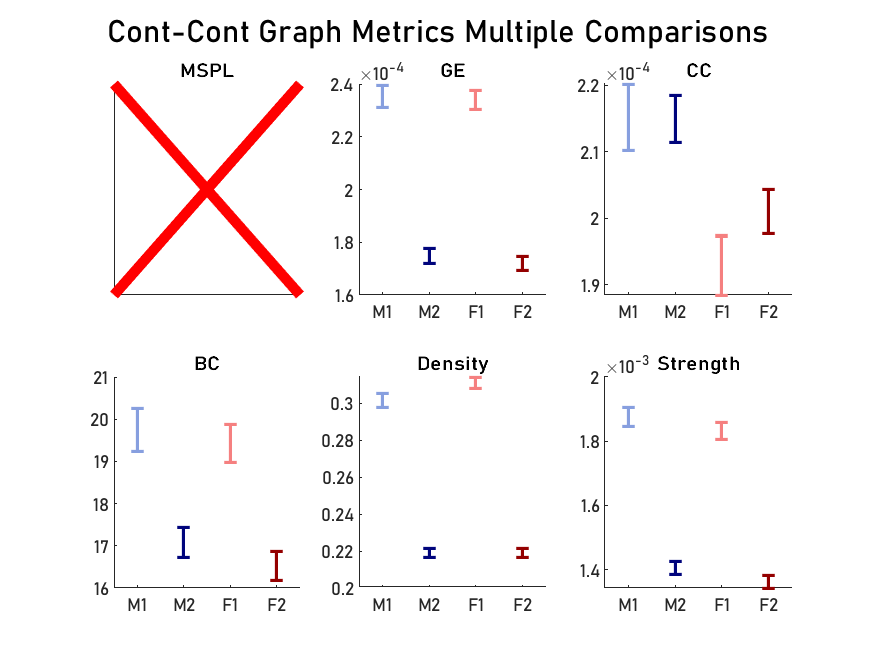


| Cont-Cont Graph Metrics Multiple Comparisons (Mean ± SE) | | | | |
| --- | --- | --- | --- | --- |
| Metric | M1 | M2 | F1 | F2 |
| MSPL | NaN | NaN | NaN | NaN |
| GE | 0.000235 ± 4.02e-06 | 0.000175 ± 2.81e-06 | 0.000234 ± 3.6e-06 | 0.000172 ± 2.63e-06 |
| CC | 0.000215 ± 4.98e-06 | 0.000215 ± 3.48e-06 | 0.000193 ± 4.46e-06 | 0.000201 ± 3.26e-06 |
| BC | 19.7 ± 0.51 | 17.1 ± 0.357 | 19.4 ± 0.458 | 16.5 ± 0.334 |
| Density | 0.302 ± 0.00359 | 0.219 ± 0.00251 | 0.311 ± 0.00322 | 0.219 ± 0.00235 |
| Strength | 0.00188 ± 2.94e-05 | 0.0014 ± 2.06e-05 | 0.00183 ± 2.64e-05 | 0.00136 ± 1.93e-05 |


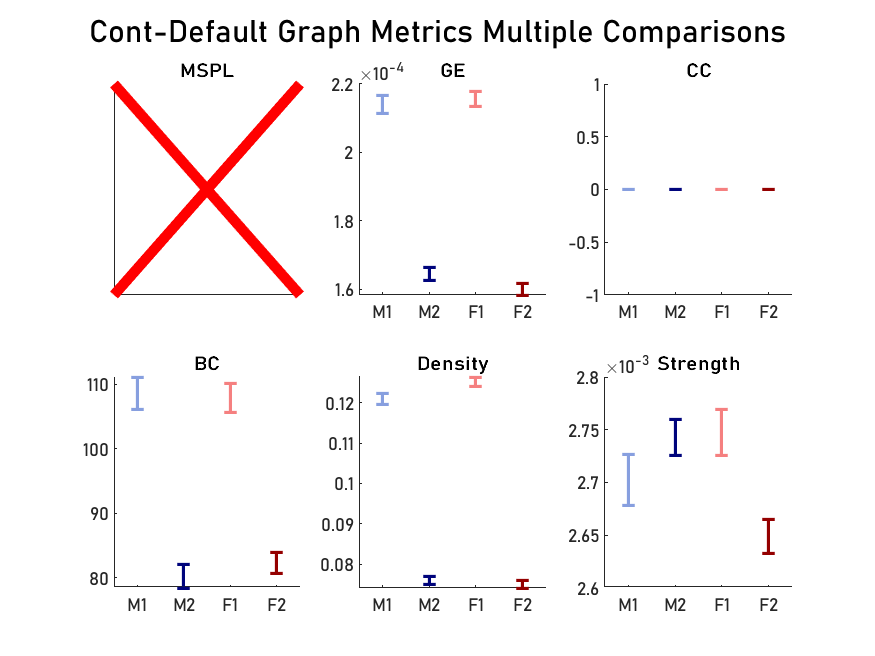


| Cont-Default Graph Metrics Multiple Comparisons (Mean ± SE) | | | | |
| --- | --- | --- | --- | --- |
| Metric | M1 | M2 | F1 | F2 |
| MSPL | NaN | NaN | NaN | NaN |
| GE | 0.000214 ± 2.56e-06 | 0.000164 ± 1.79e-06 | 0.000216 ± 2.29e-06 | 0.00016 ± 1.67e-06 |
| CC | 0 ± 0 | 0 ± 0 | 0 ± 0 | 0 ± 0 |
| BC | 109 ± 2.56 | 80.2 ± 1.79 | 108 ± 2.3 | 82.3 ± 1.68 |
| Density | 0.121 ± 0.00134 | 0.0759 ± 0.000934 | 0.125 ± 0.0012 | 0.075 ± 0.000875 |
| Strength | 0.0027 ± 2.41e-05 | 0.00274 ± 1.69e-05 | 0.00275 ± 2.16e-05 | 0.00265 ± 1.58e-05 |


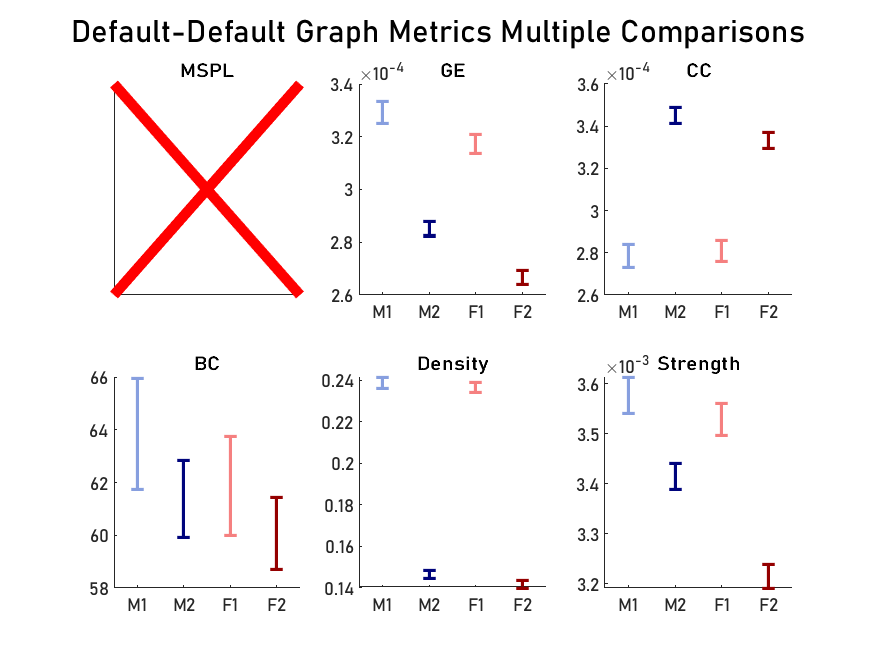


| Default-Default Graph Metrics Multiple Comparisons (Mean ± SE) | | | | |
| --- | --- | --- | --- | --- |
| Metric | M1 | M2 | F1 | F2 |
| MSPL | NaN | NaN | NaN | NaN |
| GE | 0.000329 ± 4.03e-06 | 0.000285 ± 2.82e-06 | 0.000317 ± 3.62e-06 | 0.000267 ± 2.64e-06 |
| CC | 0.000279 ± 5.48e-06 | 0.000345 ± 3.83e-06 | 0.000281 ± 4.91e-06 | 0.000333 ± 3.59e-06 |
| BC | 63.8 ± 2.09 | 61.4 ± 1.46 | 61.9 ± 1.88 | 60.1 ± 1.37 |
| Density | 0.239 ± 0.00276 | 0.146 ± 0.00193 | 0.237 ± 0.00248 | 0.142 ± 0.00181 |
| Strength | 0.00358 ± 3.61e-05 | 0.00341 ± 2.53e-05 | 0.00353 ± 3.24e-05 | 0.00321 ± 2.37e-05 |
